# Comparison of spectral and spatial denoising techniques in the context of High Definition FT-IR imaging hyperspectral data

**DOI:** 10.1038/s41598-018-32713-7

**Published:** 2018-09-25

**Authors:** Paulina Koziol, Magda K. Raczkowska, Justyna Skibinska, Sławka Urbaniak-Wasik, Czesława Paluszkiewicz, Wojciech Kwiatek, Tomasz P. Wrobel

**Affiliations:** 1 Institute of Nuclear Physics Polish Academy of Sciences, PL-31342 Krakow, Poland; 2 Faculty of Physics and Applied Computer Science, AGH University of Science and Technology, Mickiewicza 30, Krakow, Poland; 3 Faculty of Electrical Engineering, Automatics, Computer Science and Biomedical Engineering, AGH University of Science and Technology, Mickiewicza 30, Krakow, Poland; 4 NZOZ Pathology Department, Jagiellonska 70, Kielce, Poland

**Keywords:** Cheminformatics, Statistics, Imaging and sensing

## Abstract

The recent emergence of High Definition (HD) FT-IR and Quantum Cascade Laser (QCL) Microscopes elevated the IR imaging field very close to clinical timescales. However, the speed of acquisition and data quality are still the critical factors in reaching the clinic. Denoising offers aide in both aspects if performed properly. However, there is a lack of a direct comparison of the efficiency of denoising techniques in IR imaging in general. To achieve such comparison within a rigorous framework and obtaining the critical information about signal loss, a simulated dataset strongly bound by experimental parameters was created. Using experimental structural and spectral information and experimental noise levels data as an input for the simulation, a direct comparison of spatial (Fourier transform, Mean Filter, Weighted Mean Filter, Gauss Filter, Median Filter, spatial Wavelets and Deep Neural Networks) and spectral (Savitzky-Golay, Fourier transform, Principal Component Analysis, Minimum Noise Fraction and spectral Wavelets) denoising schemes was enabled. All of these techniques were compared on the simulated dataset, taking into account SNR gain, signal distortion and sensitivity to tuning parameters as comparison metrics. Later, the best techniques were applied to experimental data for validation. The results presented here clearly show the benefit of using hyperspectral denoising schemes such as PCA and MNF which outperform other methods.

## Introduction

Signal processing is a very rich field with new denoising techniques created every year. These are becoming more and more precise and adaptive in nature, however, most of them are focused on photography, medical imaging and satellite imagery^1^. Spectroscopic hyperspectral imaging in this context is relatively new and denoising techniques have not yet been fully optimized for the specific structure and characteristic of IR data^2–6^.

IR imaging provides information about the total chemical composition of the sample along with specific information about the conformation of certain molecules, e.g. proteins, DNA. This chemical contrast can be used to enable more precise models of pathology classification for many diseases, e.g. cancers, atherosclerosis or diabetes^7–12^. In recent years there has been a tremendous progress in instrumentation and image formation theory, leading to new modalities among which High Definition (HD) pushed the projected pixel size to diffraction limit sampling^13^. Obtaining the full spatial information requires using a 1.1 µm projected pixel size, which is smaller than more commonly utilized 5.5 µm one. This leads to a 25-times smaller sampling volume and light throughput, resulting in a reduction of signal to noise ratio (SNR). This significantly increases the acquisition times in order to obtain reasonable SNR in HD FT-IR and calls for denoising techniques^14^.

The current literature is lacking in a direct comparison of the efficiency of denoising techniques in IR imaging in general – this is especially difficult since every approach risks distorting the original signal when removing noise. Since it is not possible experimentally to obtain a noise-free dataset it is impossible to measure actual signal distortion. To circumvent this problem and allow a rigorous framework for comparing denoising efficiency, including the critical information about signal loss, we created a simulated dataset, strongly bound by experimental parameters. Using actual structural and spectral information and experimental noise levels data as an input for the simulation, we enabled a direct comparison of spatial and spectral denoising schemes.

As IR imaging grew from the spectroscopic community, the natural tendency was to use standard spectral denoising techniques such as Savitzky-Golay^15^ or Fourier-filtering^16^. More advanced approaches are based on Principal Component Analysis (PCA)^17^ and may take a form of Minimum Noise Fraction (MNF)^18,19^. Up to date, spatial denoising in IR imaging has been rarely used with a few exceptions of Fourier filtering^16^. This stems from the fact that IR imaging is a relatively high SNR technique and unless used in HD-IR mode with a thermal source it provides data of reasonable quality. The emergence of HD-IR has opened the need for efficient denoising algorithms^20–22^. The developments in Quantum Cascade Laser Microscopes^23^ are enabling Discrete Frequency imaging and that puts even more interest in denoising techniques based on a limited number of frequencies, i.e. spatial denoising^3^. Median, Mean and Gauss filters^1^ are commonly used in data processing of imaging data, while wavelets have been suggested as very promising techniques for IR as well^24^. Finally, Artificial Neural Networks can be used for denoising^25^ and recently the first Deep Neural Network (DNN) denoising approach was enabled in Matlab packages and is applied here to IR data for the first time.

All of these techniques were compared on the simulated dataset taking into account SNR gain, signal distortion, sensitivity to tuning parameters and influence of specific FT-IR data structure with different pixel sizes of 1.1 and 5.5 µm representing HD and standard definition imaging options. The results presented here create a benchmark comparison and facilitate the choice of denoising methods for any FT-IR dataset, which reduces the optimization required to be done by the end user.

## Materials and Methods

### Experimental

A paraffin embedded serial pancreatic tissue microarray sections were obtained from US Biomax, Inc. 5 µm thick section of the TMA was placed on a BaF_2_ salt plate for transmission IR imaging. The section was deparaffinized using a 24 h hexane bath. FT-IR measurements were performed using a Bruker Vertex70v Spectrometer in transmission mode coupled to Hyperion 3000 microscope equipped with an MCT FPA 64 × 64 detector and 15x and 36x objectives, giving pixel projections of 2.7 microns and 1.1 microns, respectively. 5.5 microns image was obtained by 5x binning of the corresponding 36x image and served as an example of typical IR imaging projected pixel size. The images were acquired in the range from 3900 cm^−1^ to 900 cm^−1^ with the resolution of 4 cm^−1^. A subset of the core, i.e. a single 64 × 64 pixels tile, was scanned with both 15x and 36x objectives with 2, 4, 8, 16, 32, 64, 128 and 256 scans. This data was used for the experimental noise level (8 levels) estimation to be used in the simulations corresponding to the experimental number of scans.

### Simulations

A single pancreatic cancer tissue core served as an input for simulations. Fuzzy C-Means clustering was performed on the 1.1 µm image (4 scans; empty pixels were discarded) with 3 chosen classes. The spatial distribution of these classes were used for initial concentration profiles of three spectral profiles (resembling proteins, lipids and DNA/RNA profiles), each containing 16 bands (1030, 1080, 1172, 1242, 1280, 1342, 1400, 1462, 1550, 1650, 1754, 2352, 2850, 2920, 2962, 3300 cm^−1^). Each band was simulated as a 50:50% mixture of Gaussian and Lorentzian peak shapes. The heights, band positions and widths were all randomized separately for each of the pixels using a normal distribution with 5%, 0, 2%-1, 8% and 1% variability, respectively, simulating experimental variability of components and concentrations. Afterward, a Gaussian filter was used for each of the variables in the spectral range, leading to simulating the dependence of IR resolution from the wavelength of light. The result of simulation was over 700 000 spectra arranged spatially in a tissue core structure, with an additional 600 000 empty space spectra. Raw signal was corrupted by randomized baseline and various levels of multiplicative noise (up to 40% for the most intense bands as reported^4^), to model different SNR levels of experimental data.

Performance of each denoising method in the group of spectral and spatial denoising techniques was evaluated based on two metrics representing data quality and distortion. For spectral denoising, data improvement was represented by Signal to Noise Ratio (SNR) with a signal value of unity and a standard deviation (after slope correction) of 2150–2080 cm^−1^ region (free of absorption bands) as a noise measure. Each SNR was divided by the SNR of a noisy signal to yield SNR Gain. Second parameter – Signal Distortion (SD), was defined as a sum of differences between denoised and clean (simulated) spectrum, but only for variables where the difference is higher than noise added to a clean signal and is presented as the total signal %. Examination of spatial denoising was carried out based on commonly used in image processing parameters, such as Peak Signal to Noise Ratio (pSNR) and Structural Similarity Index (SSIM). Similarly to SNR, pSNR takes the ratio of squared maximum value and Mean Square Error (MSE) between input and reference image. Since pSNR is usually defined on a decibel scale it was transformed into a linear scale and then divided by the corresponding transformed pSNR of the noisy signal to yield pSNR Gain. SSIM index is a multiplicative combination of the structure, contrast and luminance term^26^, giving the level of similarity to the reference image.

Data processing and classification were done in OPUS and Matlab software using built-in and home written procedures.

## Results and Discussion

**Figure 1:**
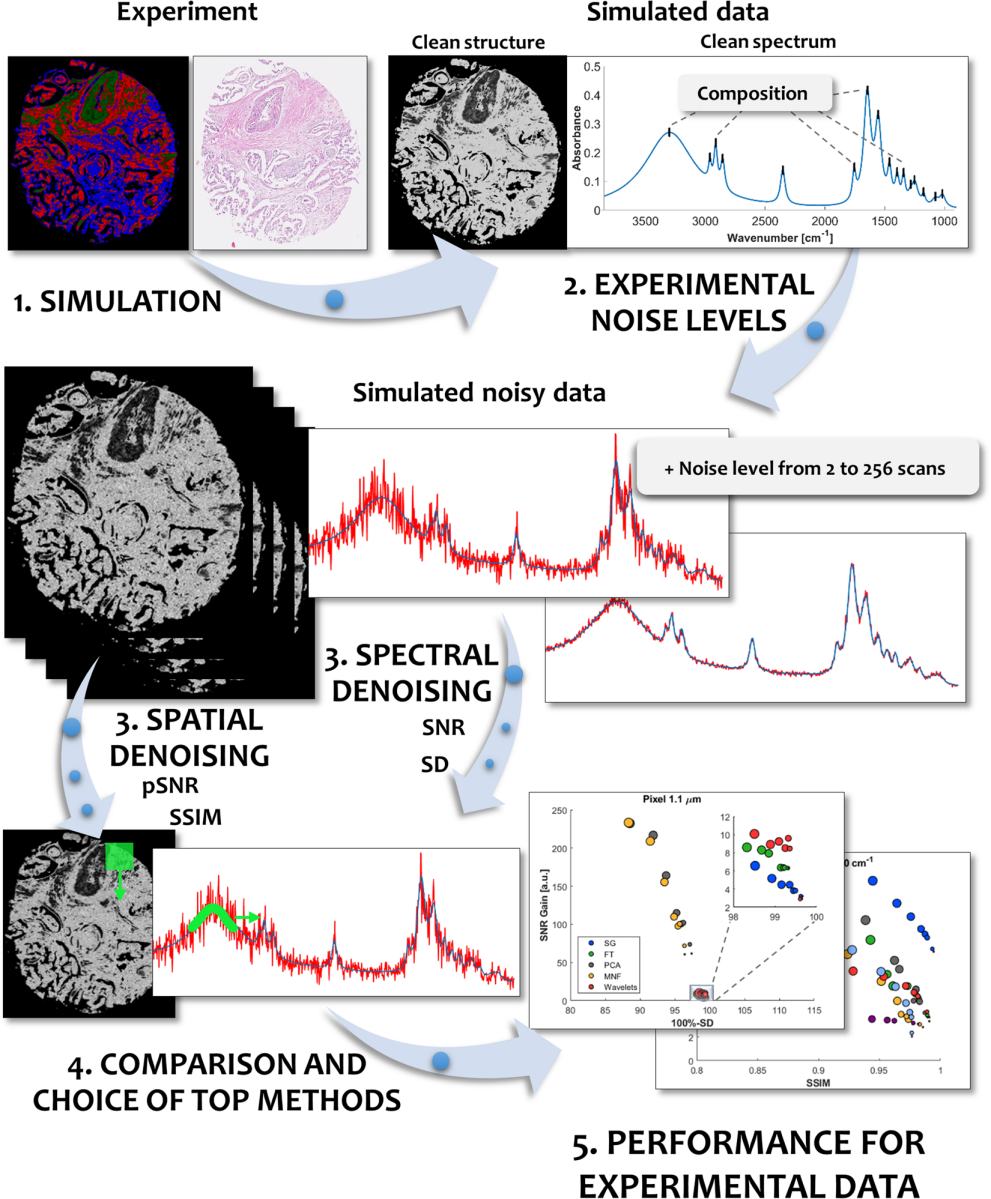
A schematic representation of the workflow designed to compare denoising techniques for IR imaging. An experimental IR image is used to define the structure and composition profiles of the dataset. Afterward, multiplicative noise is introduced to the system with levels corresponding to experimental 2 to 256 scans (8 levels). In the next step, the noisy data is subjected to different denoising approaches using spectral and spatial methods which are optimized for different noise levels. Finally, optimized methods are compared using Signal to Noise Ratio (SNR) and Signal Distortion (SD) metrics for spectral data and corresponding peak Signal to Noise Ratio (pSNR) and Structural Similarity Index (SSIM). The best methods are then applied again to experimental data to show their actual capability.

Figure 1 presents the workflow leading to a much more precise comparison of a range of denoising techniques. Experimental data served as an input for defining the structure of the image as well as relative concentrations of major biochemical classes (proteins; lipids; carbohydrates/DNA/RNA) and their spectral profiles. This allowed simulating a noise-free dataset with variability levels comparable or higher than that of a typical tissue sample. A noise-free signal was then corrupted by the addition of spectral multiplicative noise at levels corresponding to experimental values of 2 to 256 scans (8 different levels). This dataset was then subjected to spatial and spectral denoising algorithms, each optimized separately for best SNR Gain vs SD (Supplementary Materials S3–S4) and pSNR Gain vs SSIM (Supplementary Materials S5–S8). Finally, the best methods were again applied to experimental data to validate their effectiveness.

### Spectral denoising

**Figure 2:**
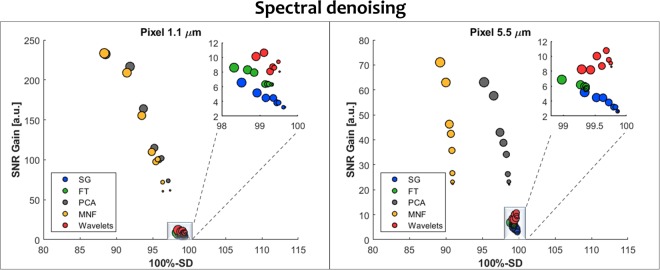
A comparison of spectral denoising techniques as measured by SNR gain (normalized to SNR of the input noisy data) and SD (presented as 100%-SD) for two different pixel sizes representing HD and standard definition imaging magnifications. Five methods were tested: Savitzky-Golay (SG), Fourier transform (FT), Principal Component Analysis (PCA), Minimum Noise Fraction (MNF) and spectral wavelets (Wavelets). The size of the dots corresponds to the initial noise level, starting with the highest noise for the largest dot (2 scans) and going down to the smallest (256 scans). The optimal parameters for each of the methods and noise levels are given in Supplementary Materials.

**Figure 3:**
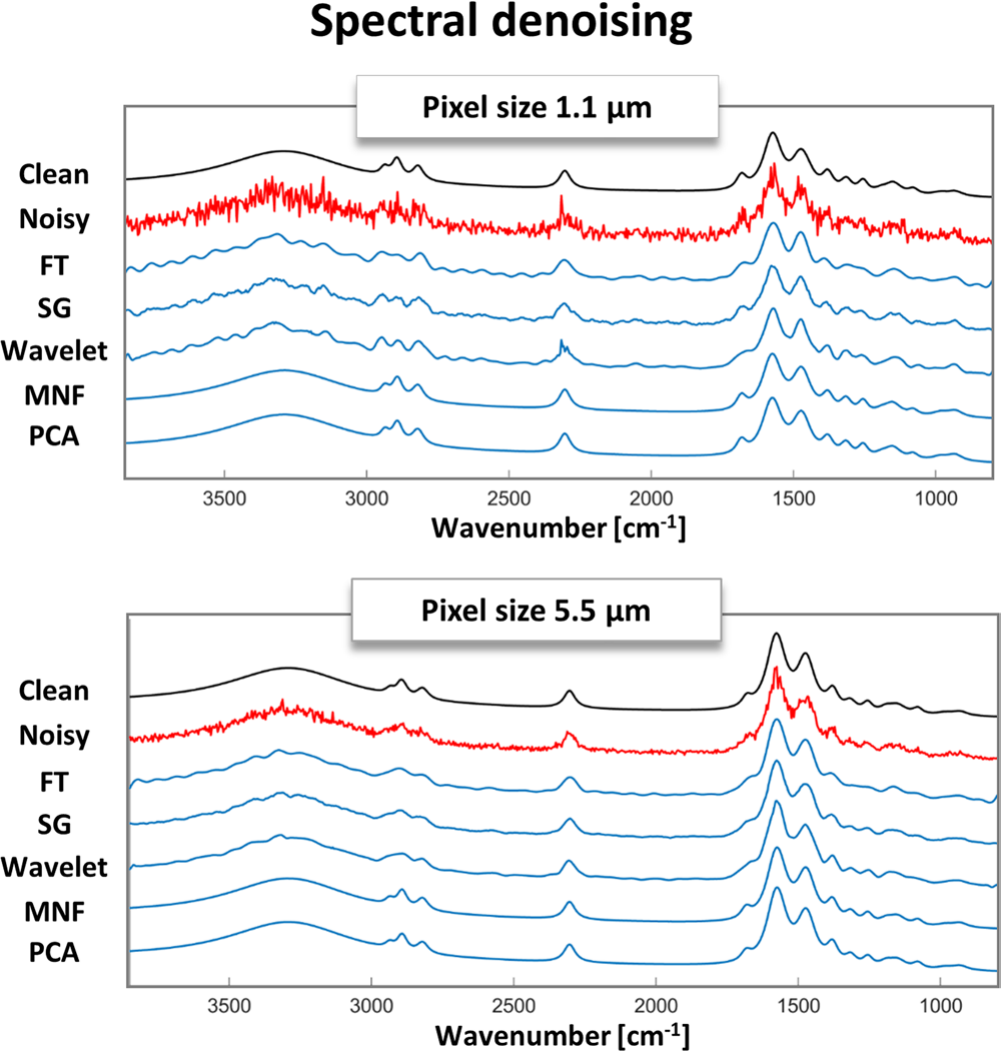
Comparison of the spectral result of denoising for a single pixel at noise levels of 2 scans for HD and standard definition modes – noisy spectrum in red and denoised spectrum in blue.

The results of SNR gain and SD for all of the spectral methods tested are shown in Fig. 2 and single pixel spectra are shown in Fig. 3. The best SNR Gain is usually obtained for the noisiest data for a given technique. The most commonly used in spectroscopy denoising algorithm is the Savitzky-Golay smoothing filter (blue) is giving the smallest SD values in the range of 0.5–1.5% at a reasonable increase of SNR of up to 6 times in the HD resolution (with optimized parameters of polynomial degree equal to 3 and the smoothing points equal to 15 and 17). The higher the initial noise level the higher the gain, but more signal was lost. The increase could be higher if the user would be satisfied with higher SD which are related in a nice, almost linear fashion (Supplementary Materials S3–S4). The next method, the Fourier transform, is showing an increased SNR gain behavior at a slightly higher cost of SD. On the downside, it is more sensitive to optimization parameters having a well-defined maximum for a given noise level (Supplementary Materials S3–S4). Wavelet denoising is also based on Fourier filter and different shapes (families) can be used with different levels of hardness, leading to 450 combinations that were tested (Supplementary Materials Tables 1 and 2). Since the best wavelets significantly flattened the spectra, Pearson correlation coefficient was used to support the choice of the best set. The latter performed better than Savitzky-Golay and Fourier transform in terms of SNR gain having values of 8–10 with SD at a comparable level, however, significant optimization was required and the choice of the best ones is somewhat arbitrary. The methods discussed up to now work on a single spectrum level, while the next two methods take advantage of the multivariate nature of the data using the whole data-cube with thousands of spectra at once. PCA denoising performs a matrix decomposition of the whole dataset at once and MNF has an additional step of noise whitening before the same matrix decomposition as PCA. This additional step provides an estimate of the noise structure and helps to order the new directions in space in decreasing SNR. For HD data both PCA and MNF outclass every other method with the SNR gain in the range of 60 to 240 times, however, this comes at a cost of larger SD values in the 3–12% range. Moreover, the choice of optimal parameters is not obvious, since the more PCA or MNF bands are used for reconstruction the less signal is lost but the SNR Gain drops (Supplementary Materials S3–S4).

**Figure 4:**
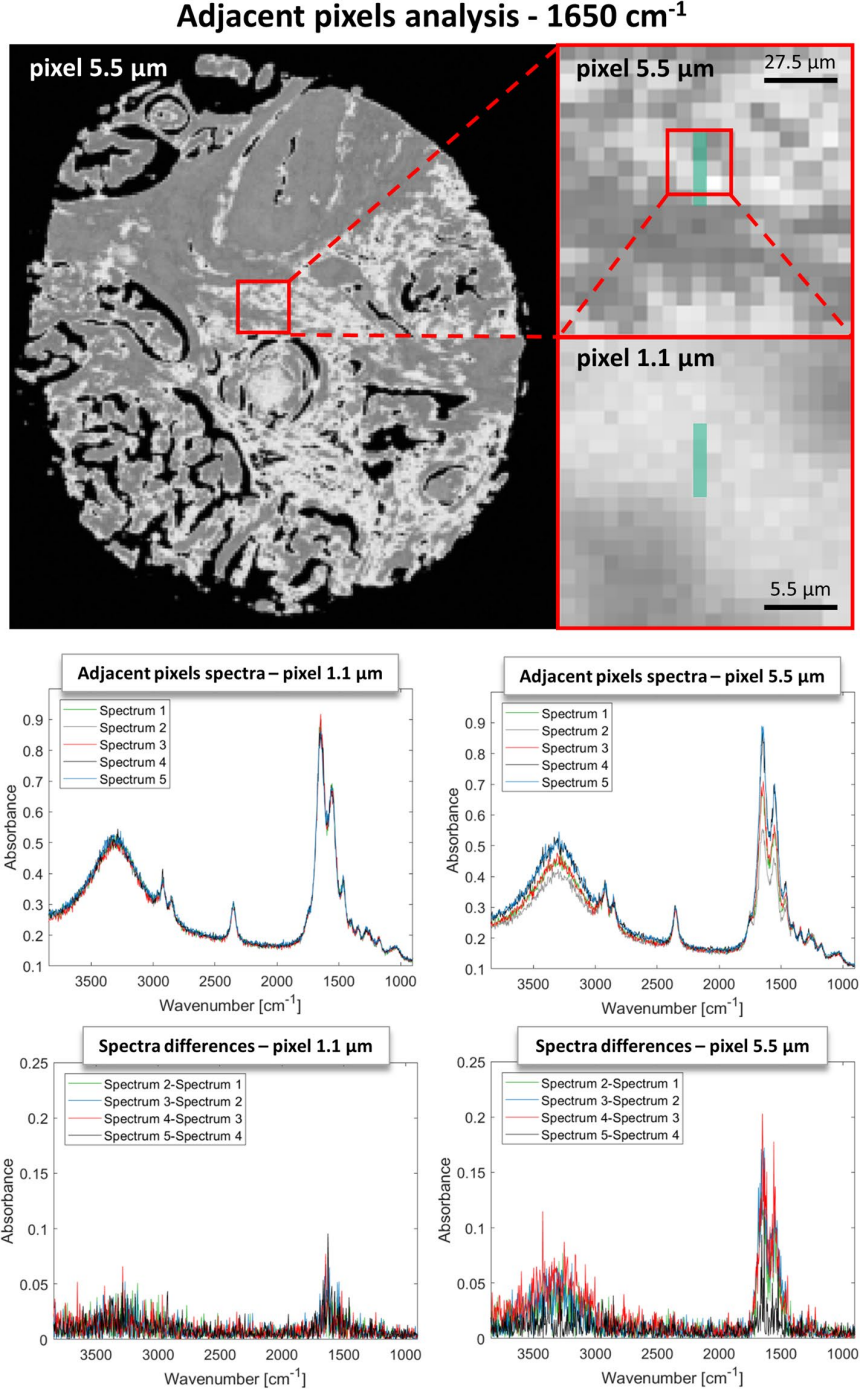
Example of calculation of the shift difference stat in MNF. The spectra of 5 adjacent pixels and their differences in 1.1 µm and 5.5 µm image of the same area are shown.

The situation for standard definition imaging at 5.5 µm is quite similar, however, the starting noise level is already smaller than in HD and the SNR gains are also smaller. Savitzky-Golay, Fourier transform and Waveletes have slightly smaller SNR gains than in HD, but compensating in smaller SD values. PCA performs in a similar fashion as in HD, however, MNF has a significantly higher SD, which is almost constant at around 10%, regardless of the noise level. This is a bit surprising but can be understood by analyzing the way the noise estimate is obtained. A shift difference stat is most commonly used, which calculates the difference between adjacent pixels, assuming that the only difference is noise. Results of such calculation and this effect are shown in Fig. 4.

The differences between adjacent pixels show a presence of the signal in both projected pixel size images. However, the assumption about only noise being present is met in HD data to a greater extent than in standard definition. The origin of this is the different spatial sampling of the actual structure and the fact, that HD is point-spread function limited and standard definition is not. This phenomenon is moreover wavelength dependent since a wavelength of 6 µm will be better sampled with 5.5 µm pixels that a wavelength of 3 µm. This leads to higher signal loses at the high-wavenumber end of the spectra than in the fingerprint and can be seen in Fig. 3, where exemplary single pixel spectra are shown before and after denoising. Interestingly, this is not lowered with the inclusion of more MNF bands as happens in PCA.

### Spatial denoising

**Figure 5:**
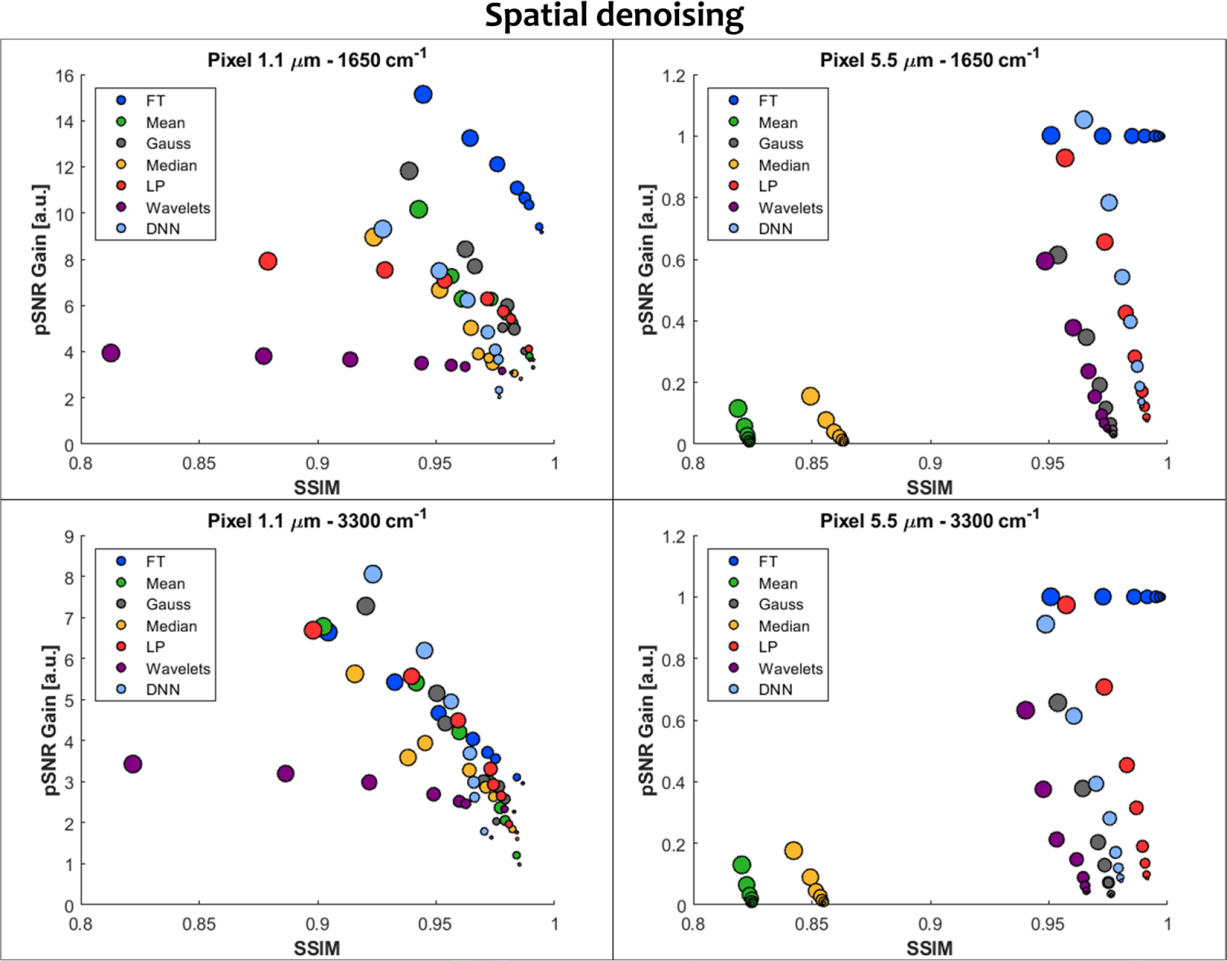
A comparison of spatial denoising techniques as measured by pSNR Gain (normalized to pSNR of the input data) and SSIM for two different pixel sizes representing HD and standard definition imaging magnifications and two spectral bands of 3300 cm^−1^ and 1650 cm^−1^, corresponding to 3 and 6 µm wavelengths. Seven methods were tested: Fourier transform (FT), Mean Filter (Mean), Gauss Filter (Gauss), Median Filter (Median), Weighted Mean (W. Mean), spatial Wavelets (Wavelets) and Deep Neural Networks (DNN). The size of the dots corresponds to the initial noise level, starting with the highest noise for the largest dot (2 scans) and going down to the smallest (256 scans). The optimal parameters for each of the methods and noise levels are given in Supplementary Materials.

Spatial denoising was performed with 7 different approaches and the results are summarized in Fig. 5. In general, the wavelength dependence of the image structure and contrast coming from spatial resolution required to focus on two examples – 3300 cm^−1^ and 1650 cm^−1^ bands, corresponding to 3 and 6 µm and having a different contrast. For HD imaging case the lowest pSNR gains were obtained for wavelets and surprisingly also the highest signal loss (low SSIM values) for both bands. This means that wavelets are not properly estimating the structure of human tissue sections as in photography this approach with modifications is very efficient. For the 1650 cm^−1^ band Fourier transform outperformed all other methods. This comes from the fact that image at this wavelength is highly oversampled and the corresponding structure frequency is significantly different from the noise frequency. The pSNR gains range from 9 to 16 with a very small signal loss as measured by SSIM. The 3300 cm^−1^ band results show that most methods perform similarly, however, for the highest noise levels Deep Neural Networks obtained the highest pSNR gain. The standard definition imaging counterpart shows interesting results, i.e. that most of the methods decrease pSNR values – the exception being Fourier transform. Deeper investigation of this surprising result shows that again the structure sampled as 5.5. µm vary highly from pixel to pixel causing mixing of the structure signal with the noise component. This leads to signal removal and is not recommended for standard definition data. Profiles of the structure before and after denoising showing this effect are presented in Supplementary Materials (Figure S11).

**Figure 6:**
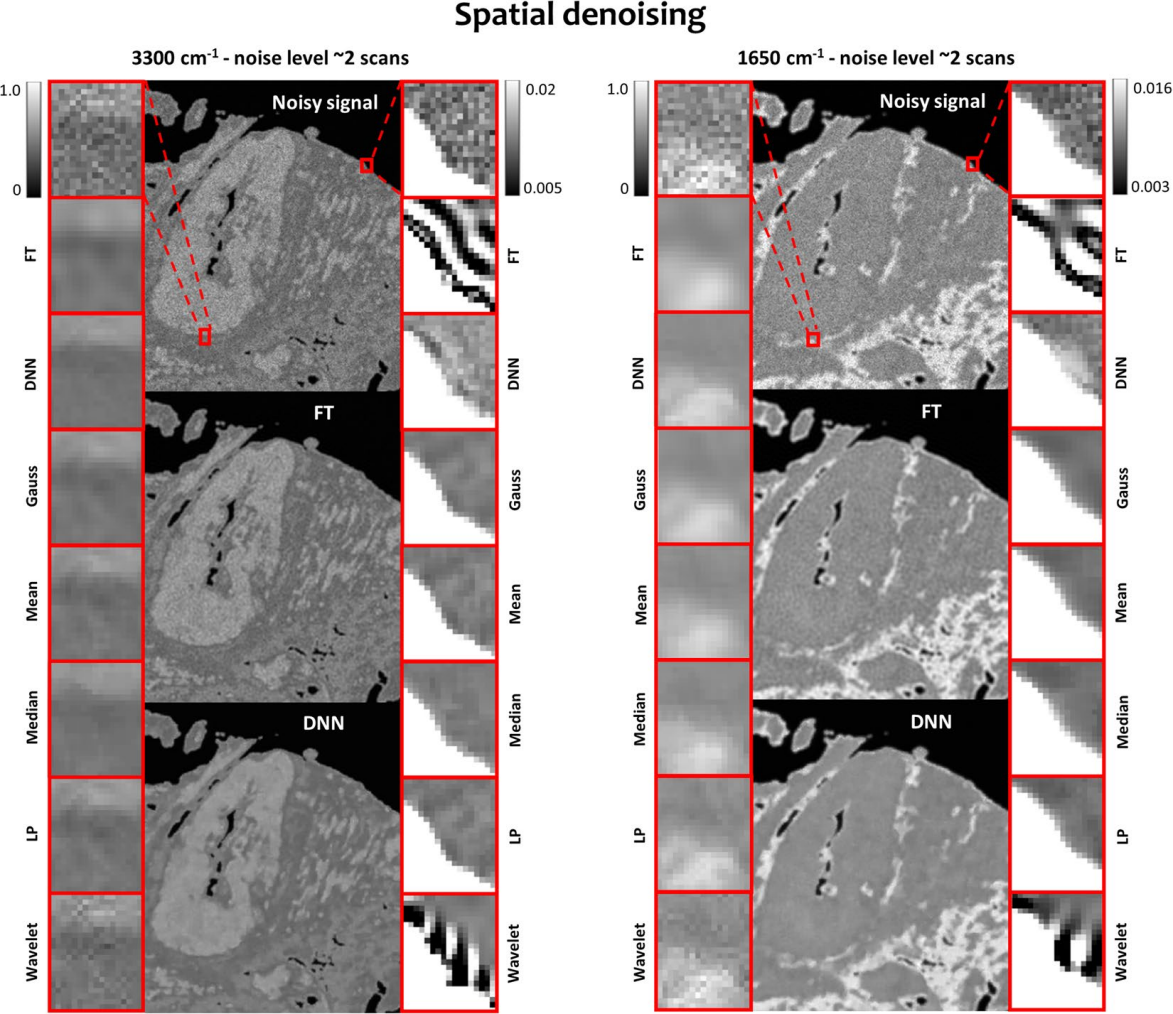
Details of spatial denoising results for HD data with Fourier transform (FT) and Deep Neural Networks (DNN) shown in details as they performed relatively the best (all images are given in Supplementary Materials S10). Zoom-ins on tissue structure and edges are shown to better highlight noise rejection and potential artifacts introduced by a given method.

pSNR and SSIM are very popular metrics for estimating the efficiency of denoising methods in the signal processing community, they are not, however, without drawbacks. The HD images before and after denoising for the all of the methods are shown in Fig. 6. It can be seen that even though Fourier transform had the best values for pSNR and SSIM it leads to artifact creation at the edges – an expected feature of this denoising method. Wavelets as methods based on Fourier transform share a similar artifact, however, to a smaller degree. Human eye suggests that the Deep Neural Network actually performs better than Fourier transform and Mean, Median, W. Mean and Gauss filters are quite comparable.

Interestingly, when pSNR and SSIM were calculated for the same variables after spectral PCA and MNF denoising, the results are in the order of 33 and 113 of pSNR gain and 0.95–0.99 for SSIM for PCA and 37 to 30 pSNR gain and 0.96–0.97 for SSIM for MNF. This indicates that these spectral methods perform better spatially than any other spatial filter. To be exact, MNF does use spatial information about the noise structure, therefore it is not strictly spectral.

### Experimental data denoising

**Figure 7:**
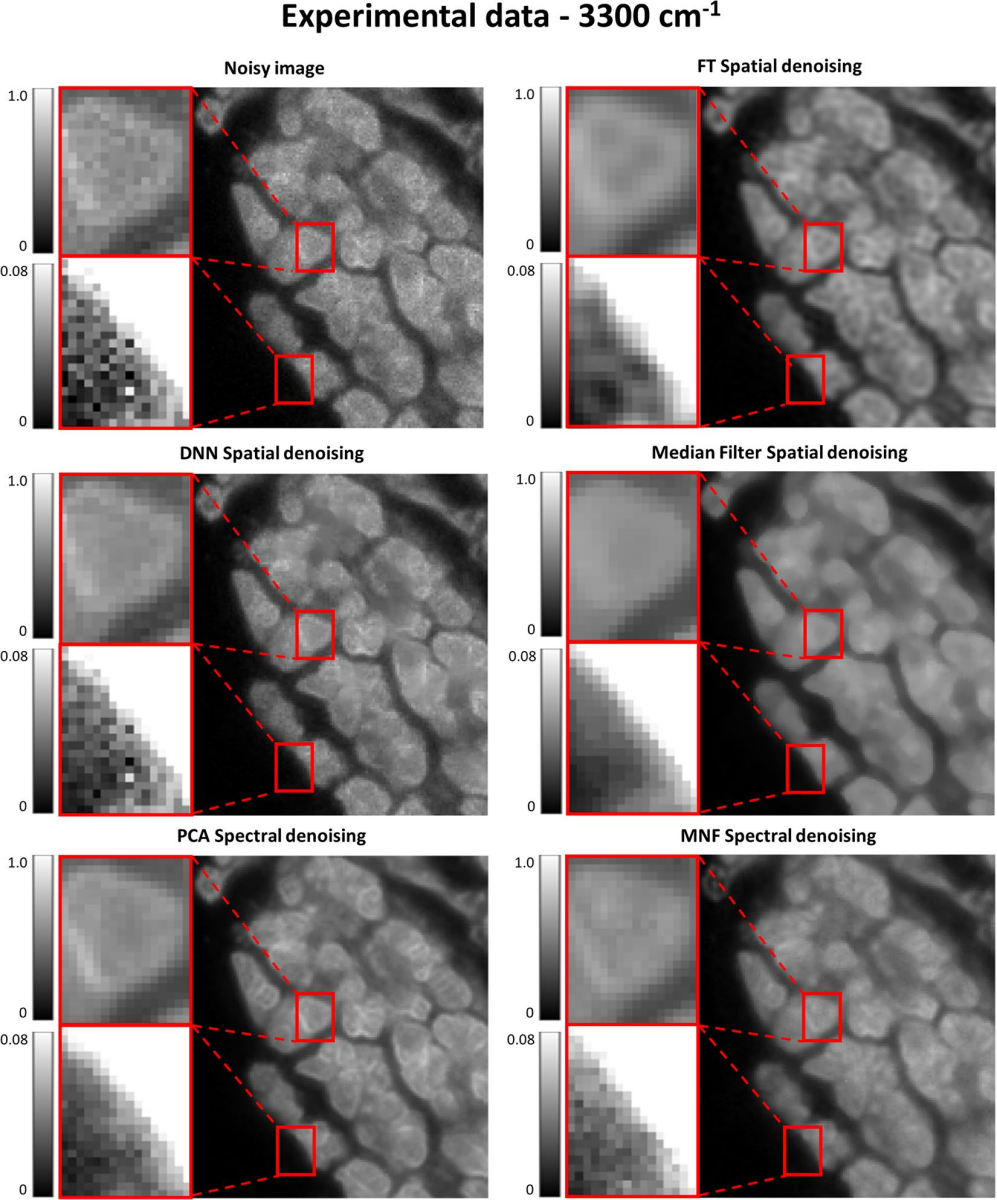
Results of spatial and spectral denoising on the image quality of a healthy pancreatic tissue sample. Median, FT, DNN, PCA and MNF denoising methods were applied to an experimental image acquired with 4 scans.

**Figure 8:**
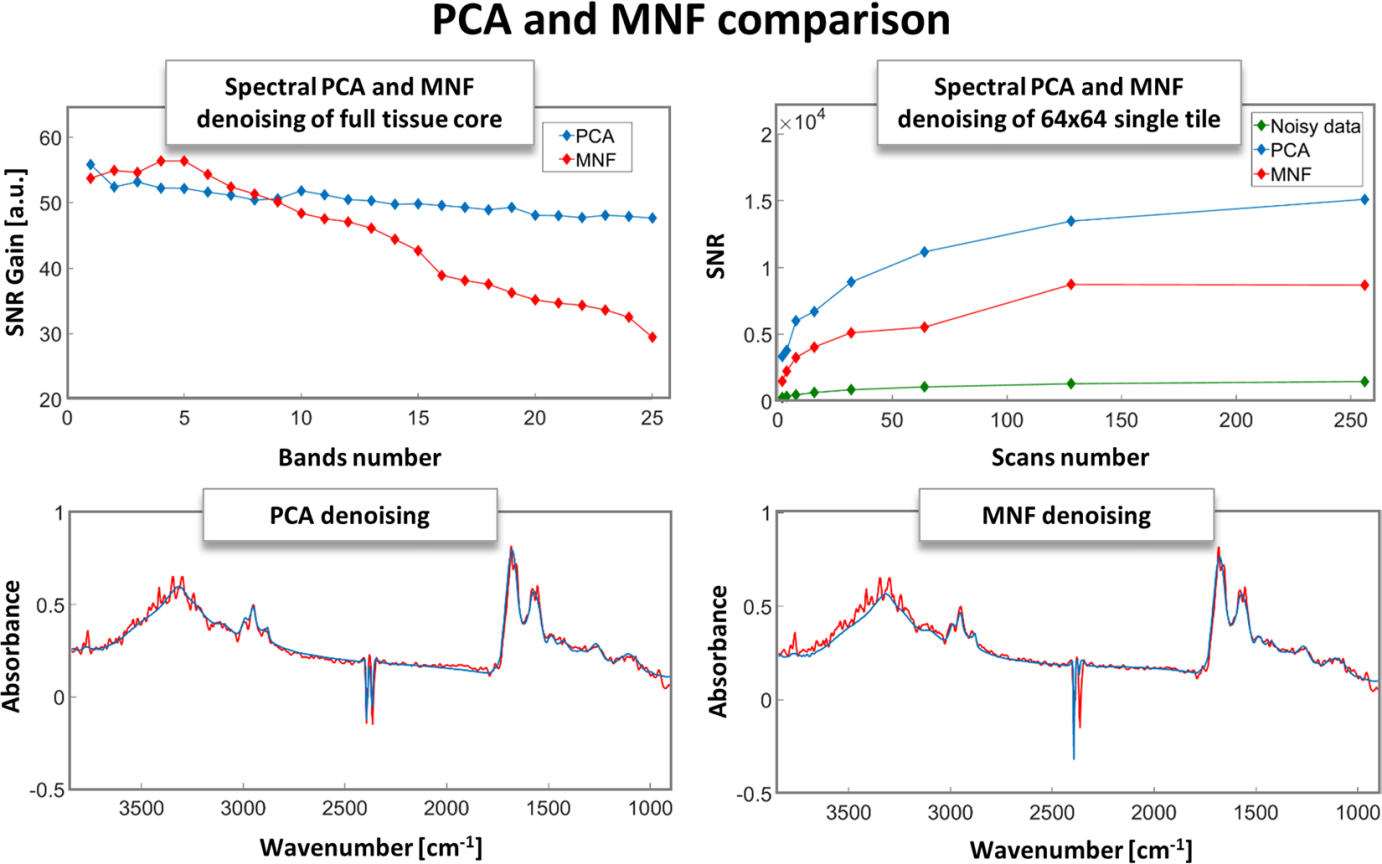
(Upper left) Comparison of SNR Gains for PCA and MNF denoising with a different number of bands taken for reconstructions for an experimental whole tissue core shown in Fig. 7. (Bottom row) Single spectra taken from the same experimental pixel from Fig. 7. (Upper right) Actual SNR values of an experimental 2 to 256 scans of a single 64 × 64 tile of a tissue compared with PCA and MNF denoised values of the same image, showing the clear gain from denoising regardless of the actual number of scans.

Every simulation is burdened by an assumption which may or may not be met in real life and therefore, it is always important to verify theoretical findings with experimental data. A healthy pancreatic tissue core was used for this purpose and the results of the best denoising methods are shown in Figs 7 and 8. It can be seen that a popular Median Filter is quite good at reducing noise, but significantly distorts the structure especially at the edges. FT Spatial Filter on the hand preserves more structural detail but leads to blurring and an impression of defocusing. Deep Neural Network provides a milder noise removal at the gain of preservation of the original structure. The balance between noise removal and structure preservation is apparent here. The multivariate methods of PCA and MNF do seem to perform the best, with PCA actually highlighting some edges more than can be seen in the original image. This comes from structure propagation from other spectral bands that influence the result as well. MNF also performs very well, however, edges do get smeared out due to the way the noise estimate is calculated as was explained in the simulation section.

Image quality is important, however, the most useful information coming from IR imaging is the spectral information about the chemical content. It is this information that is used for understanding changes occurring in the sample and is the basis for almost all disease classification approaches bed on IR imaging. Both PCA and MNF outperformed every other technique in both spectral and spatial denoising efficiency and a closer comparison on experimental data was done. The same pancreatic tissue core, which fragment is shown in Fig. 6 was reconstructed using a different number of bands (MNF bands and PCA loadings) and the resulting SNR Gain was calculated and is presented in Fig. 8. The overall SNR Gain is in the 30–60 range, unlike the simulation, where it reached over 200. This can be explained by a non-perfect simulation model and confirms the need to test experimental data. Still, the SNR gain is very high and drops with the incorporation of more bands, which is expected since consecutive bands will have an increasing noise component. It can be seen that MNF is more efficient at ordering the bands according to SNR, while PCA uses variance as a criterion for ordering. Up to band 10, MNF reconstructions have a better SNR gain and outperform PCA. Interestingly, the SNR Gain drops much faster if too many bands are taken for reconstruction, while PCA remains relatively constant. Therefore, if properly optimized MNF will yield a better SNR but for an unknown signal PCA is safer. Overall, both methods are yielding very good results as can be seen by the single spectra. The differences between PCA and MNF reconstructed spectra can be seen in the high wavenumber region, where MNF has an offset with regards to the experimental signal. Also, the CO_2_ bands at 2300–2400 cm^−1^ are reconstructed in a slightly different way, showing that some of the signal loss is irrelevant since these bands are not of use in interpretation. Finally, when looking at SNR plot for the different number of scans it is clear that no matter how many scans are taken experimentally, denoising will improve it beyond the experimentally reasonable limits. This measurement was done on a smaller area, therefore, the SNR Gain is smaller than for a full tissue core, where much more pixels are available for noise estimation and results in lower MNF performance than that of PCA. A summary of the pros and cons of the different techniques is given in Table 1.

**Table 1:** A summary of the tested methods with a description of their performance.

Spectral denoising technique	PROS	CONS
Savitzky-Golay	Very low signal distortion, short computational time	SNR gain lower than one order of magnitude, two parameters optimization
Fourier-Transform	Very low signal distortion, easy to implement, short computational time, easy to optimize	SNR gain lower than one order of magnitude
PCA	Significant SNR gain and reasonable signal distortion	Medium difficulty algorithm, time and memory consuming computations
MNF	Significant SNR gain and reasonable signal distortion	Difficult algorithm, hard to implement, time and memory consuming computations
Wavelets	Very low signal distortion	SNR gain around one order of magnitude, time consuming calculation and optimization
**Spatial denoising technique**	**PROS**	**CONS**
Fourier-Transform	Good pSNR gain, high SSIM, easy to implement, reasonable computation time, easy to optimize	Image artifacts
Mean Filter	Good SSIM, easy to implement, low computational time	Mild pSNR gain
Median Filter	Good SSIM, easy to implement, low computational time	Mild pSNR gain
Gauss Filter	Good SSIM, easy to implement, low computational time	Mild pSNR gain
Weighted Mean Filer	Good SSIM, easy to implement, low computational time	Mild pSNR gain
Wavelets	Noticeable pSNR gain	Low pSNR gain and SSIM, time consuming calculation and optimization, image artifacts
Deep Neural Networks	Reasonable pSNR and SSIM	Difficult algorithm to train

## Conclusions

We investigated the denoising efficiency and signal distortion properties of a series of spectral and spatial noise removal techniques, using HD FT-IR data as an input. The results clearly show that multivariate based techniques of PCA and MNF outperform any other spatial or spectral method, taking full advantage of the broadband nature of the data. For single pixel level denoising, Wavelet denoising performed the best, while for a single frequency spatial denoising the answer was less clear, but Deep Neural Networks performed very well across different inputs and suggest that this method has good potential if trained properly. Overall, we show that any kind of denoising done on even a small number of scans for imaging will give a better outcome than extended acquisition times.

## Electronic supplementary material

Supplementary materials
