# Author Correction: Comparison of spectral and spatial denoising techniques in the context of High Definition FT-IR imaging hyperspectral data

**DOI:** 10.1038/s41598-020-62740-2

**Published:** 2020-03-25

**Authors:** Paulina Koziol, Magda K. Raczkowska, Justyna Skibinska, Sławka Urbaniak-Wasik, Czesława Paluszkiewicz, Wojciech Kwiatek, Tomasz P. Wrobel

**Affiliations:** 1 Institute of Nuclear Physics Polish Academy of Sciences, PL-31342 Krakow, Poland; 2 Faculty of Physics and Applied Computer Science, AGH University of Science and Technology, Mickiewicza 30, Krakow, Poland; 3 Faculty of Electrical Engineering, Automatics, Computer Science and Biomedical Engineering, AGH University of Science and Technology, Mickiewicza 30, Krakow, Poland; 4 NZOZ Pathology Department, Jagiellonska 70, Kielce, Poland

Correction to: *Scientific Reports* 10.1038/s41598-018-32713-7, published online 25 September 2018

This Article contains errors due to a mistake made in one of the Matlab scripts written for Signal Distortion (SD) parameter calculations, resulting in erroneous values of SD for PCA and MNF denoising methods.

As a result, in the Results and Discussion section, under the subheading ‘Spectral Denoising’,

“For HD data both PCA and MNF outclass every other method with the SNR gain in the range of 60 to 240 times, however, this comes at a cost of larger SD values in the 3–12% range. Moreover, the choice of optimal parameters is not obvious, since the more PCA or MNF bands are used for reconstruction the less signal is lost but the SNR Gain drops (Supplementary Materials S3–S4).”

should read:

“For HD data both PCA and MNF outclass every other method with the SNR gain in the range of 60 to 240. This also comes with a very little cost of SD values in the 0.5-1.6% range (comparable to other techniques). Although, the choice of optimal parameters is not obvious, since the more PCA or MNF bands are used for reconstruction the less signal is lost but the SNR Gain drops (Supplementary Materials S3–S4).”

“PCA performs in a similar fashion as in HD, however, MNF has a significantly higher SD, which is almost constant at around 10%, regardless of the noise level.”

should read:

“PCA performs in a similar fashion as in HD, however, MNF has a significantly higher SD, which is around 6.7-8.3%, and vary slightly with the noise level.”

The correct Fig. 2 appears below as Fig. 1.

**Figure 1:**
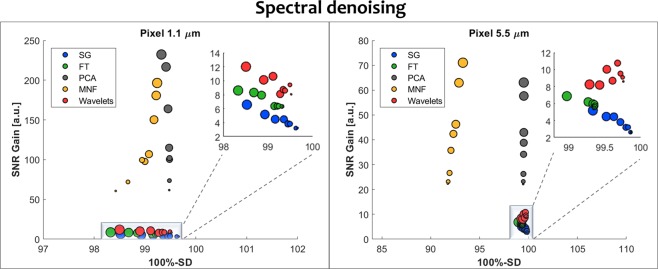
A comparison of spectral denoising techniques as measured by SNR gain (normalized to SNR of the input noisy data) and SD (presented as 100%-SD) for two different pixel sizes representing HD and standard definition imaging magnifications. Five methods were tested: Savitzky-Golay (SG), Fourier transform (FT), Principal Component Analysis (PCA), Minimum Noise Fraction (MNF) and spectral wavelets (Wavelets). The size of the dots corresponds to the initial noise level, starting with the highest noise for the largest dot (2 scans) and going down to the smallest (256 scans). The optimal parameters for each of the methods and noise levels are given in Supplementary Materials.

Furthermore, in Table 1, in the MNF Spectral denoising technique Cons,

“Difficult algorithm, hard to implement, time and memory consuming computations”

should read:

“Difficult algorithm, very hard to implement, time and memory consuming computations, signal distortion level dependent on the data structure”

Finally, the correct Supplementary Figures S3 and S4 appear below as Figs. 2 and 3 respectively.

**Figure 2:**
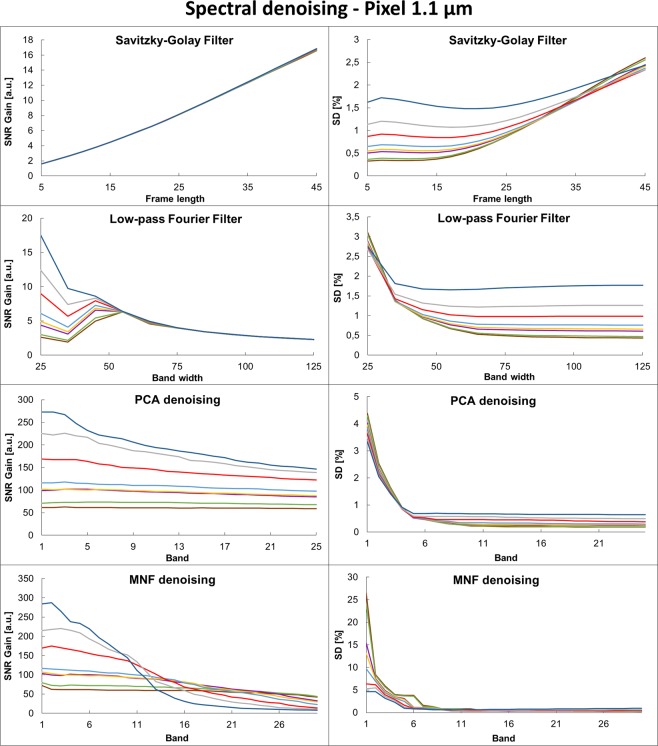
Spectral denoising parameters optimization for PCA, MNF, Low-Pass Fourier Filter and Savitzky-Golay Filter for projected pixel size of 1.1 µm and different noise levels in the range from 2 to 256 scans (legend in figure S1) with left column showing SNR Gain and SD on the corresponding graph in the right column.

**Figure 3:**
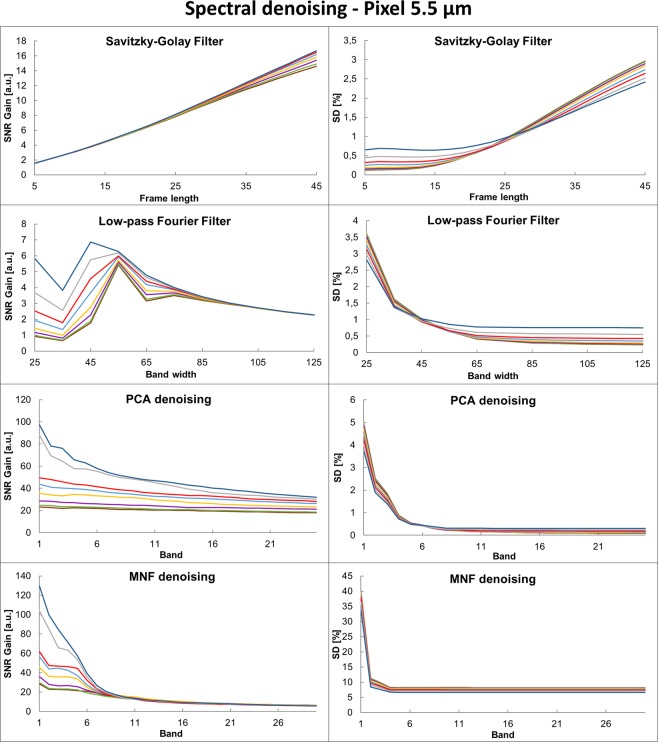
Spectral denoising parameters optimization for PCA, MNF, Low-pass Fourier Filter and Savitzky-Golay Filter for projected pixel size of 5.5 µm and different noise levels in the range from 2 to 256 scans (legend in figure S2) with left column showing SNR Gain and SD on the corresponding graph in the right column.

