# Supplementary material for: Comparison of spectral and spatial denoising techniques in the context of High Definition FT-IR imaging hyperspectral data
Source: Sci Rep. 2018 Sep 25;8:14351. doi: 10.1038/s41598-018-32713-7 (PMC6156560; doi:10.1038/s41598-018-32713-7)
Supplement: Supplementary file 1 — Supplementary materials [file 41598_2018_32713_MOESM1_ESM.docx]

**Supplementary materials:**

**Comparison of spectral and spatial denoising techniques in the context of High Definition FT-IR imaging hyperspectral data**

Paulina Koziol^1^, Magda K. Raczkowska ^1,2^, Justyna Skibinska^1,3^, Sławka Urbaniak-Wasik^4^, Czesława Paluszkiewicz^1^, Wojciech Kwiatek^1^, Tomasz P. Wrobel^1^*

1. Institute of Nuclear Physics Polish Academy of Sciences, PL-31342 Krakow, Poland

2. Faculty of Physics and Applied Computer Science, AGH University of Science and Technology, Mickiewicza 30, Krakow, Poland

3. Faculty of Electrical Engineering, Automatics, Computer Science and Biomedical Engineering, AGH University of Science and Technology, Mickiewicza 30, Krakow, Poland

4. NZOZ Pathology Department, Jagiellonska 70, Kielce, Poland

*email: [tomasz.wrobel@ifj.edu.pl](mailto:tomasz.wrobel@ifj.edu.pl)

*Optimization of each method internal parameters*

The following section shows plots of the main optimization parameter for each of the methods and the resulting SNR and SD for spectral denoising and pSNR and SSIM for spatial denoising.


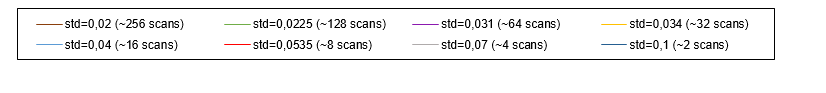


**S1**. Legend with standard deviation and corresponding multiplicative noise level for graphs presenting spectral and spatial denoising parameters optimization for projected pixel size of 1.1 µm.


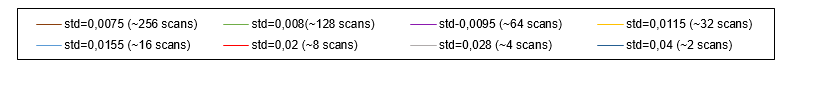


**S2**. Legend with standard deviation and corresponding multiplicative noise level for graphs presenting spectral and spatial denoising parameters optimization for projected pixel size of 5.5 µm.


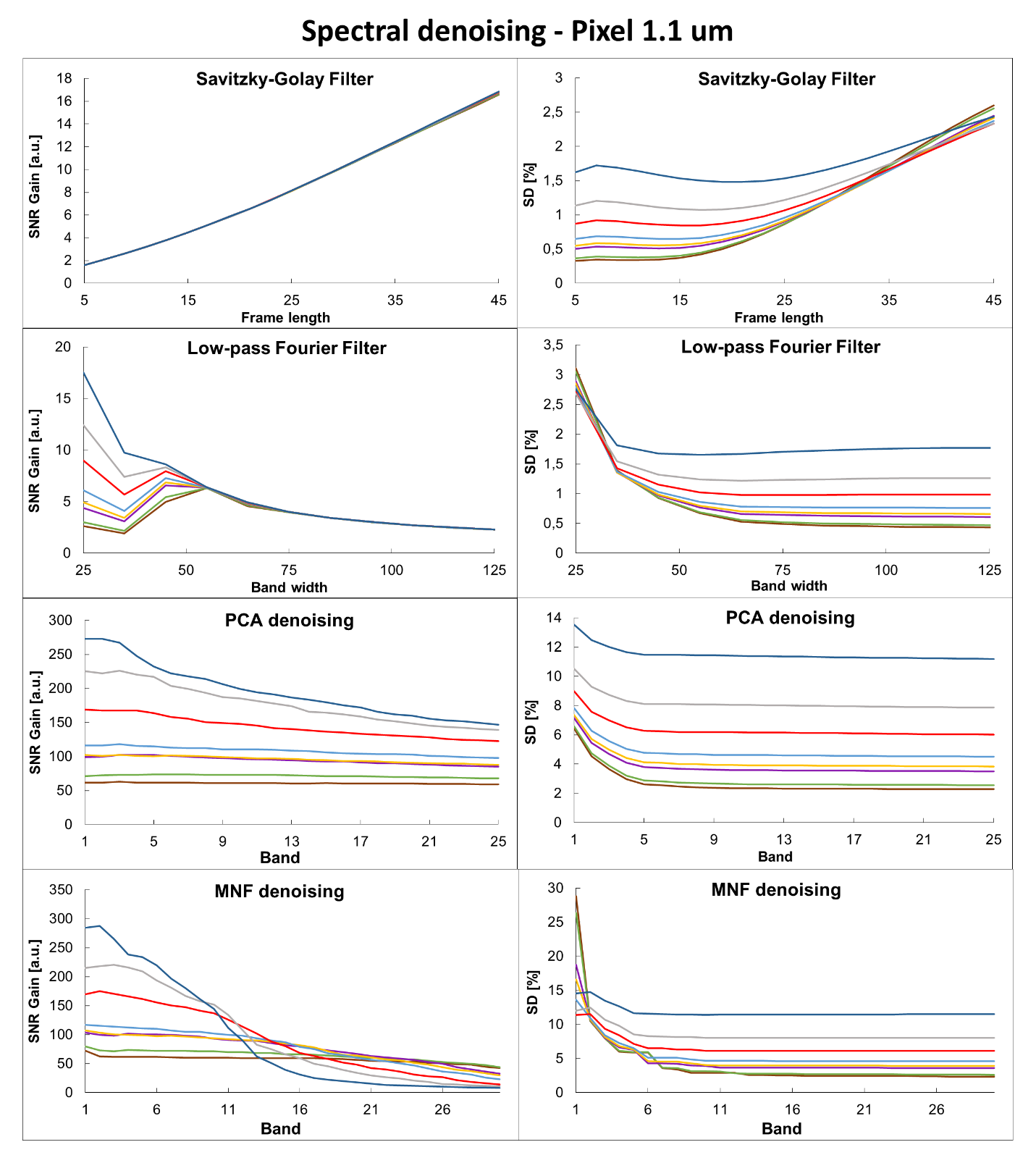


**S3**. Spectral denoising parameters optimization for PCA, MNF, Low-Pass Fourier Filter and Savitzky-Golay Filter for projected pixel size of 1.1 µm and different noise levels in the range from 2 to 256 scans (legend in figure S1) with left column showing SNR Gain and SD on the corresponding graph in the right column.


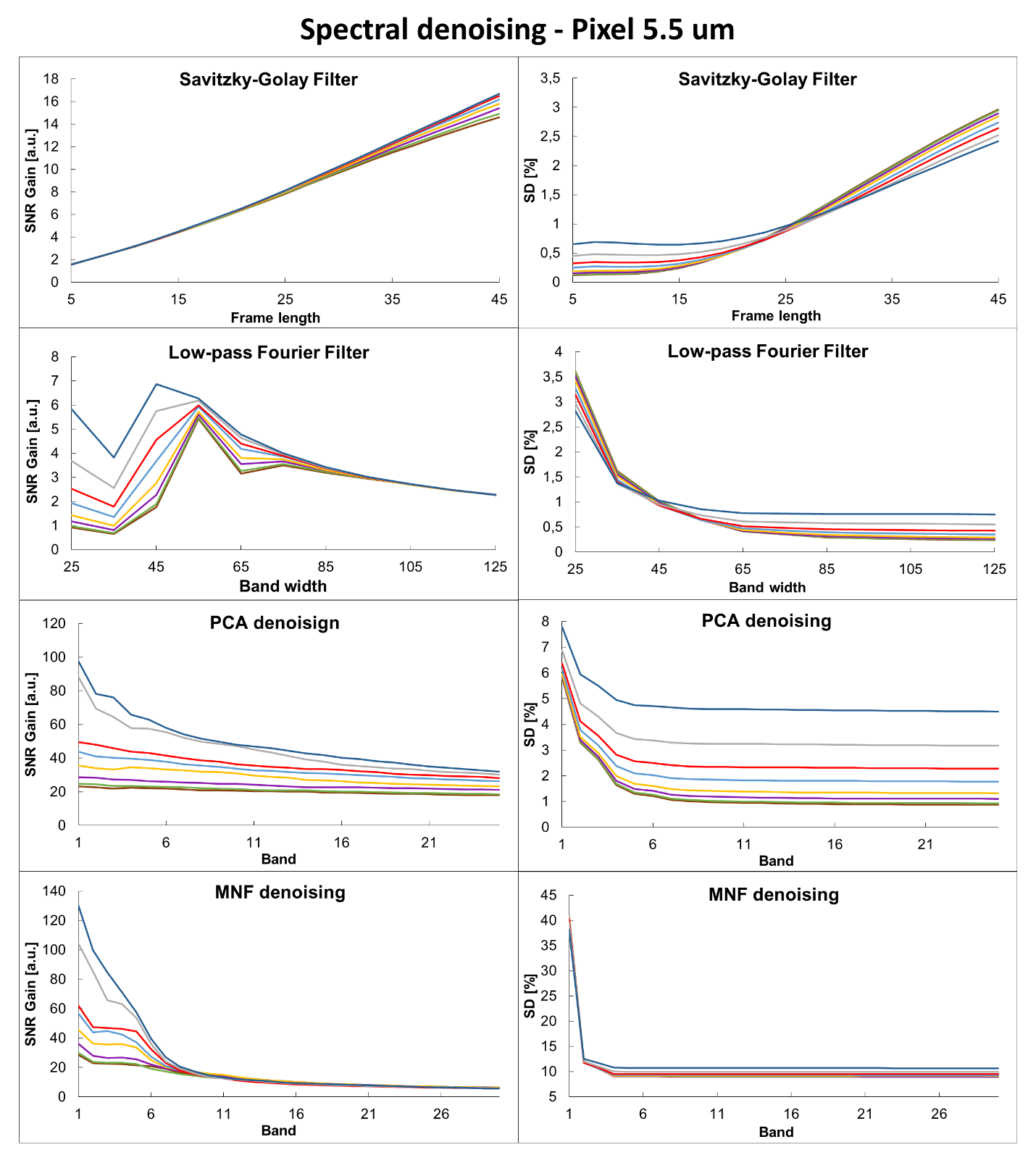


**S4.** Spectral denoising parameters optimization for PCA, MNF, Low-pass Fourier Filter and Savitzky-Golay Filter for projected pixel size of 5.5 µm and different noise levels in the range from 2 to 256 scans (legend in figure S2) with left column showing SNR Gain and SD on the corresponding graph in the right column.


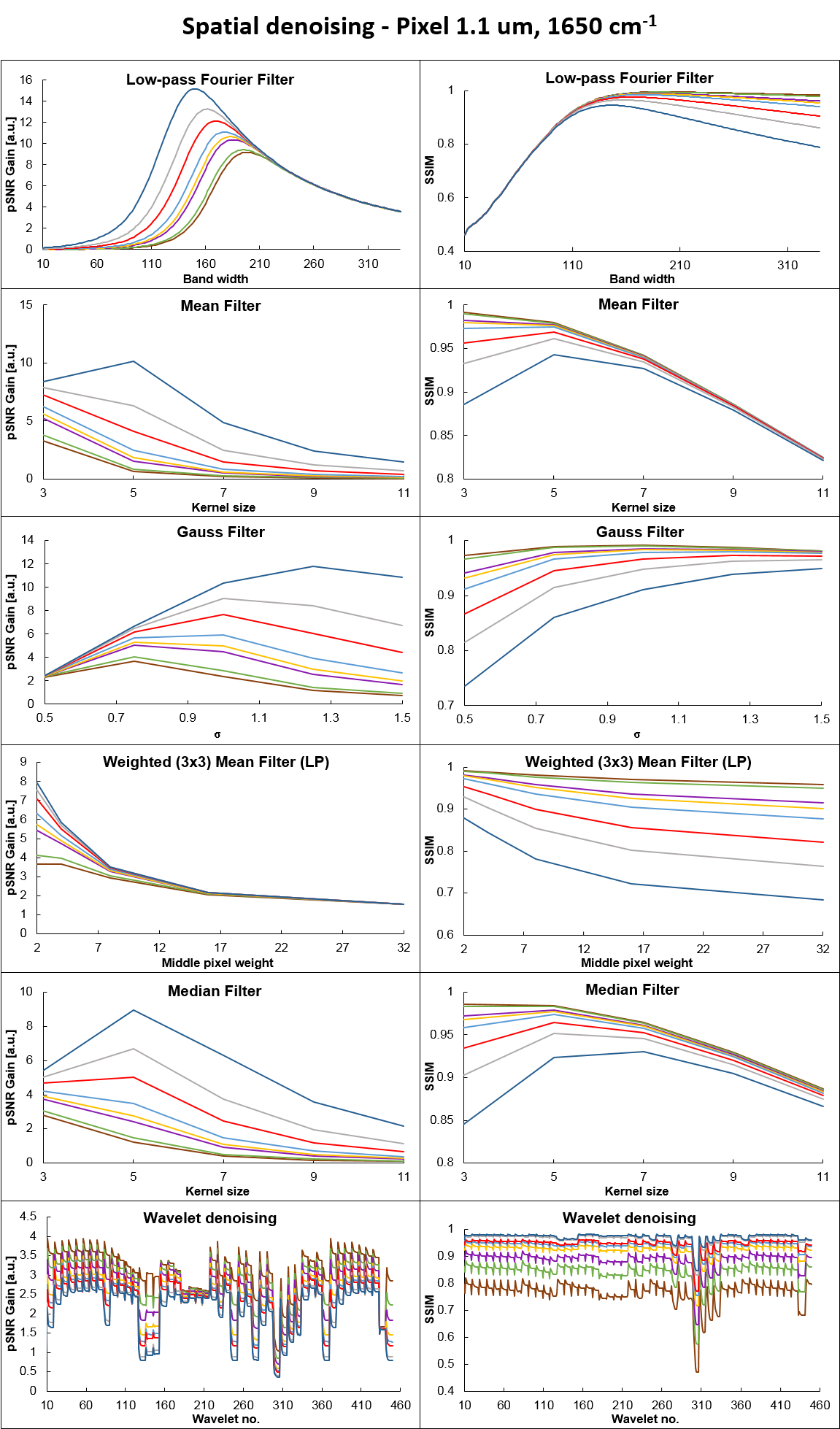


**S5.** Spatial denoising parameters optimization based on 1650 cm^-1^ image for Low-pass Fourier, Mean, Gauss, Weighted Mean, Median Filter and Wavelet denoising for projected pixel size of 1.1 µm and different noise levels in the range from 2 to 256 scans (legend in figure S1) with left column showing pSNR Gain and SSIM on the corresponding graph in the right column.


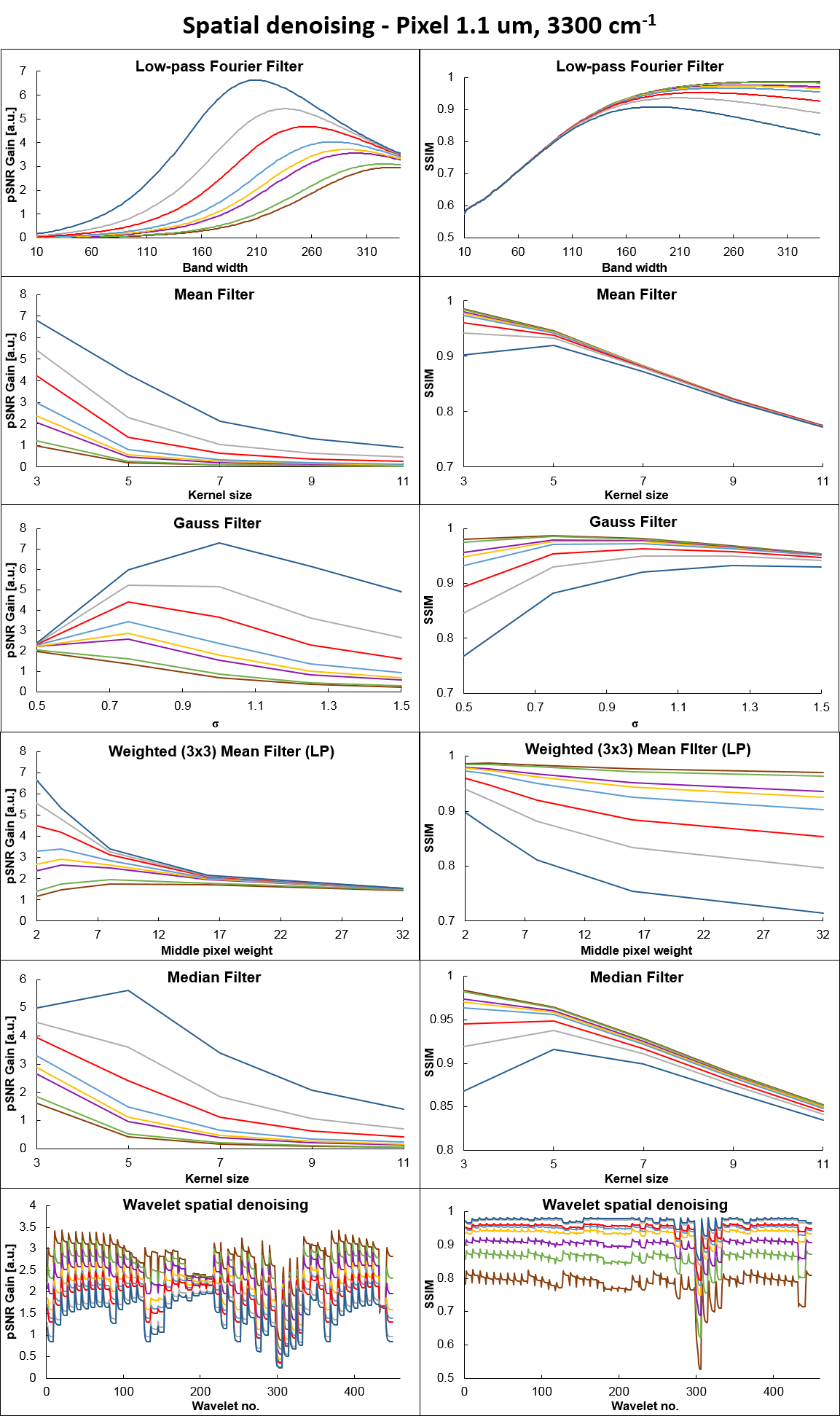


**S6.** Spatial denoising parameters optimization based on 3300 cm^-1^ image for Low-pass Fourier, Mean, Gauss, Weighted Mean, Median Filter and Wavelet denoising for projected pixel size of 1.1 µm and different noise levels in the range from 2 to 256 scans (legend in figure S1) with left column showing pSNR Gain and SSIM on the corresponding graph in the right column.


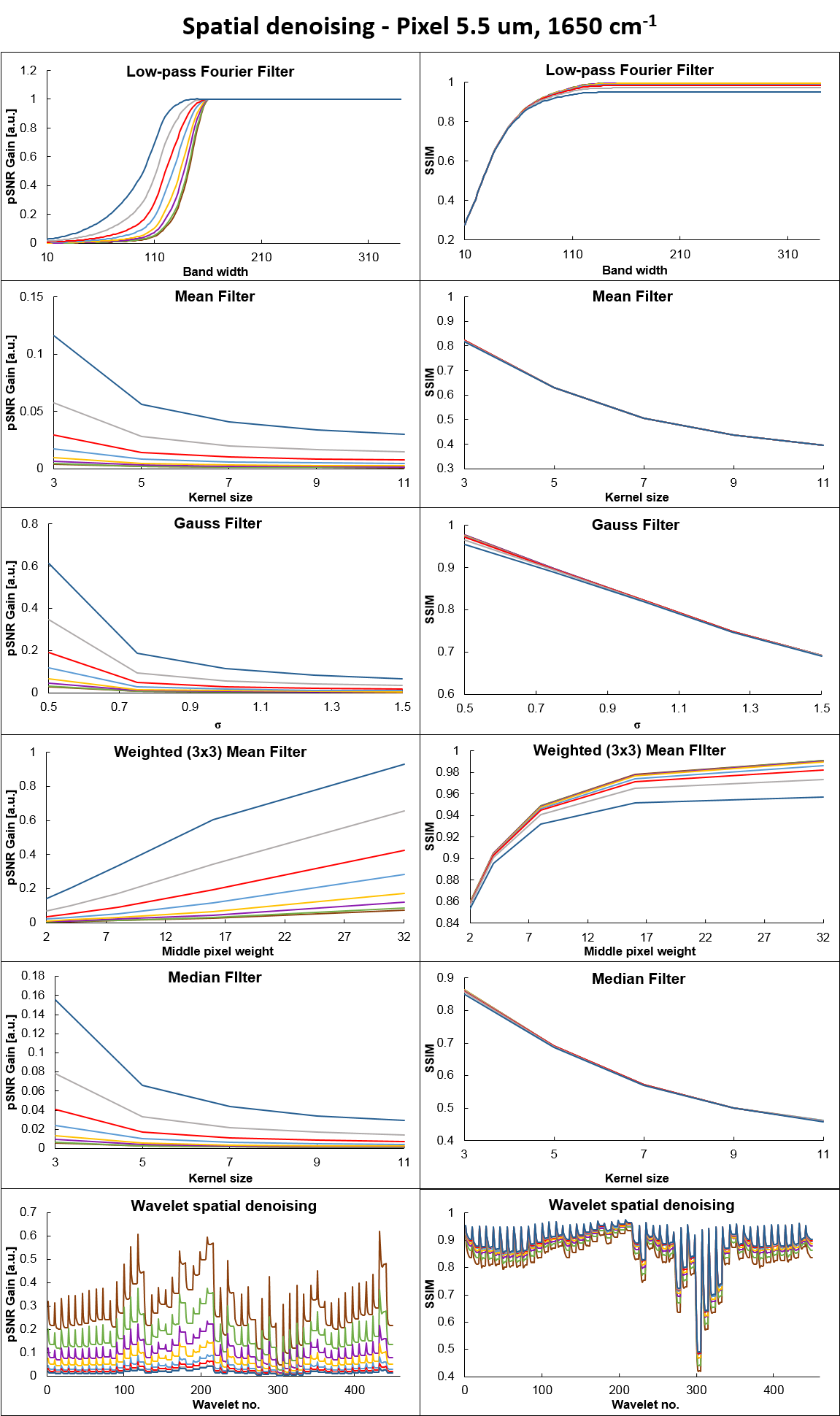


**S7.** Spatial denoising parameters optimization based on 1650 cm^-1^ image for Low-pass Fourier, Mean, Gauss, Weighted Mean, Median Filter and Wavelet denoising for projected pixel size of 5.5 µm and different noise levels in the range from 2 to 256 scans (legend in figure S2) with left column showing pSNR Gain and SSIM on the corresponding graph in the right column.


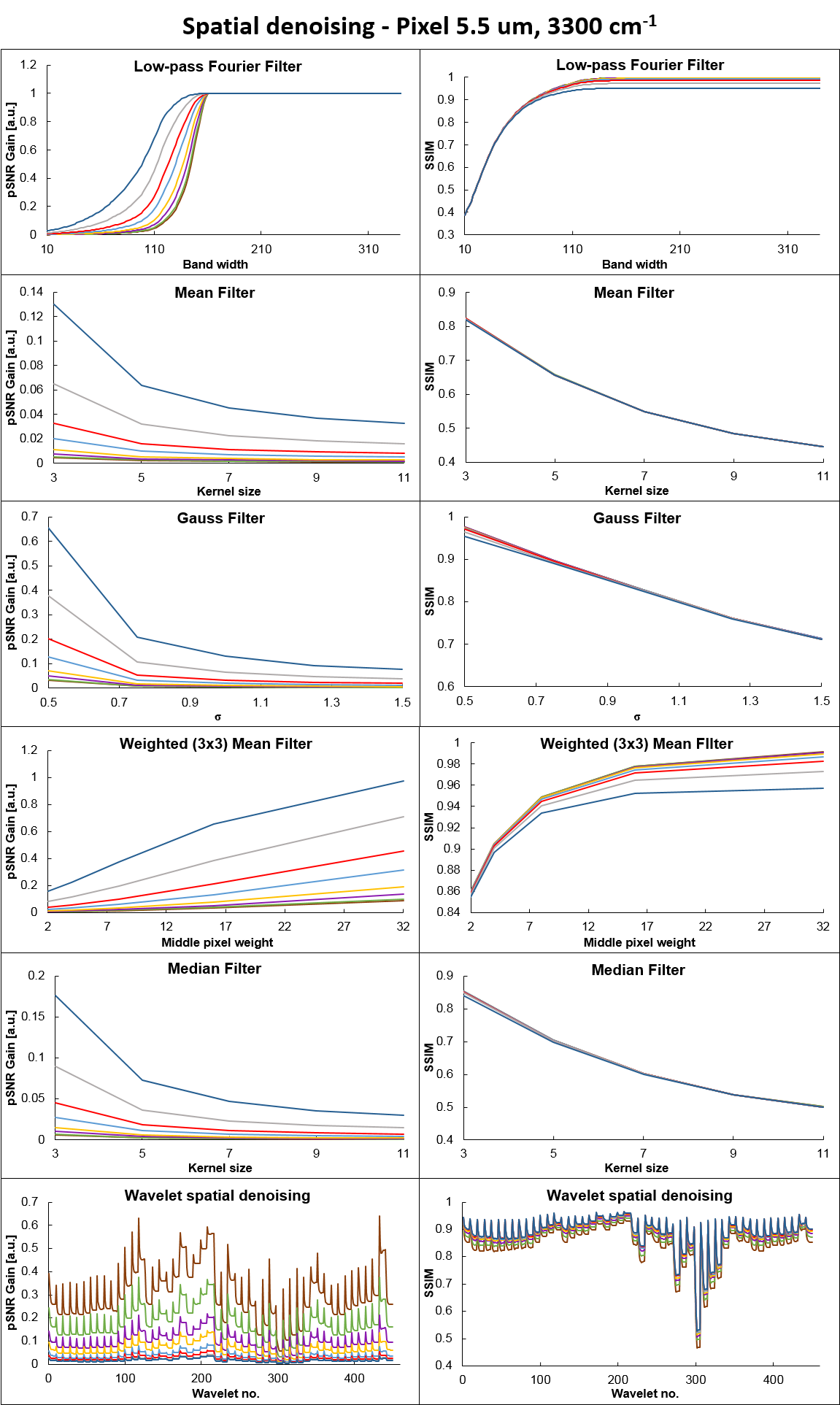


**S8.** Spatial denoising parameters optimization based on 3300 cm^-1^ image for Low-pass Fourier, Mean, Gauss, Weighted Mean, Median Filter and Wavelet denoising for projected pixel size of 5.5 µm and different noise levels in the range from 2 to 256 scans (legend in figure S2) with left column showing pSNR Gain and SSIM on the corresponding graph in the right column.

*Spatial denoising additional examples*


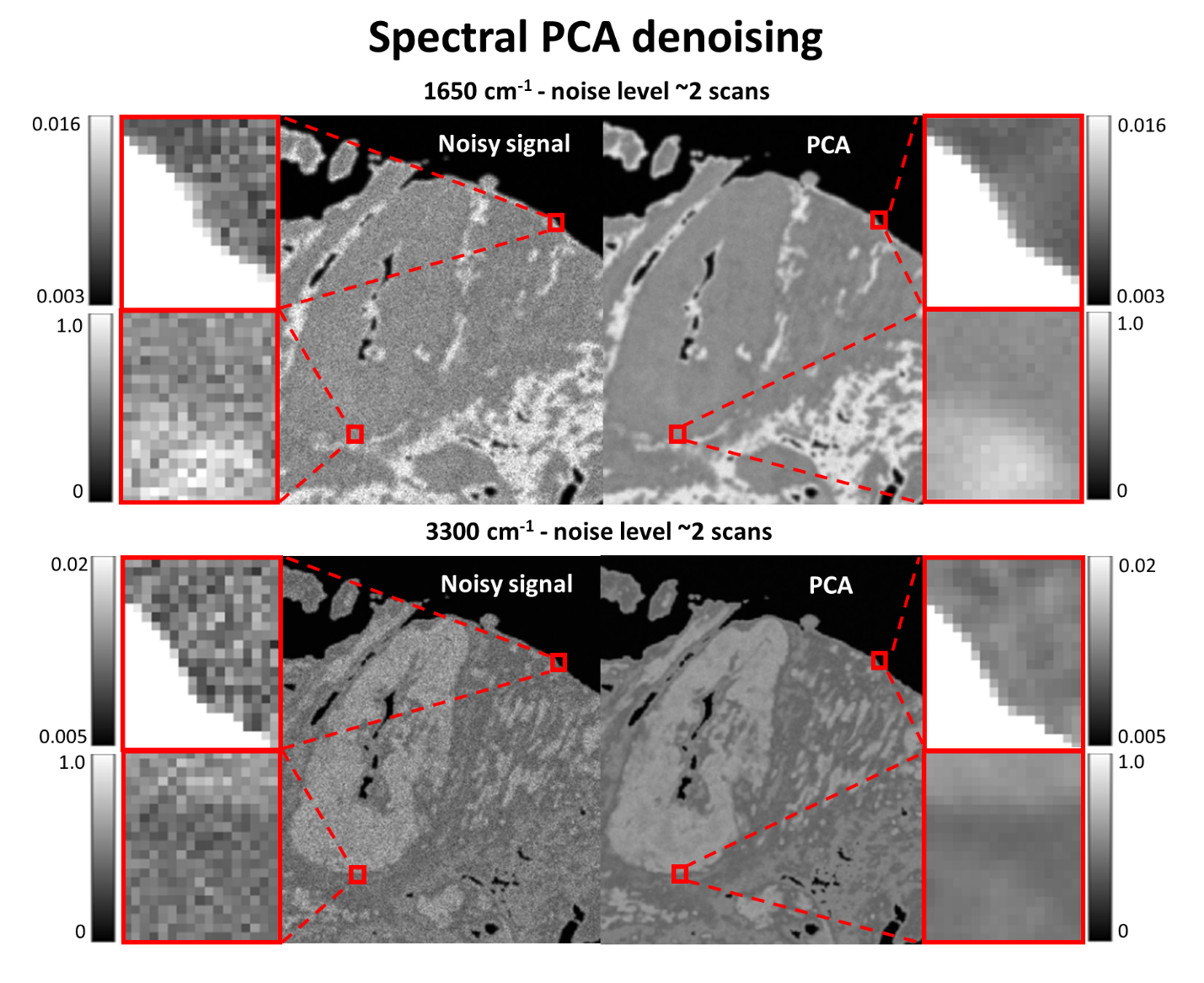


**S9.** Influence of spectral PCA denoising of simulated data on spatial noise for images 1650 cm^-1^ (upper) and 3300 cm^-1^ (bottom) and noise level of 2 scans. Zoom-ins on tissue structure and edges are shown to better highlight noise smoothening and potential artifacts.


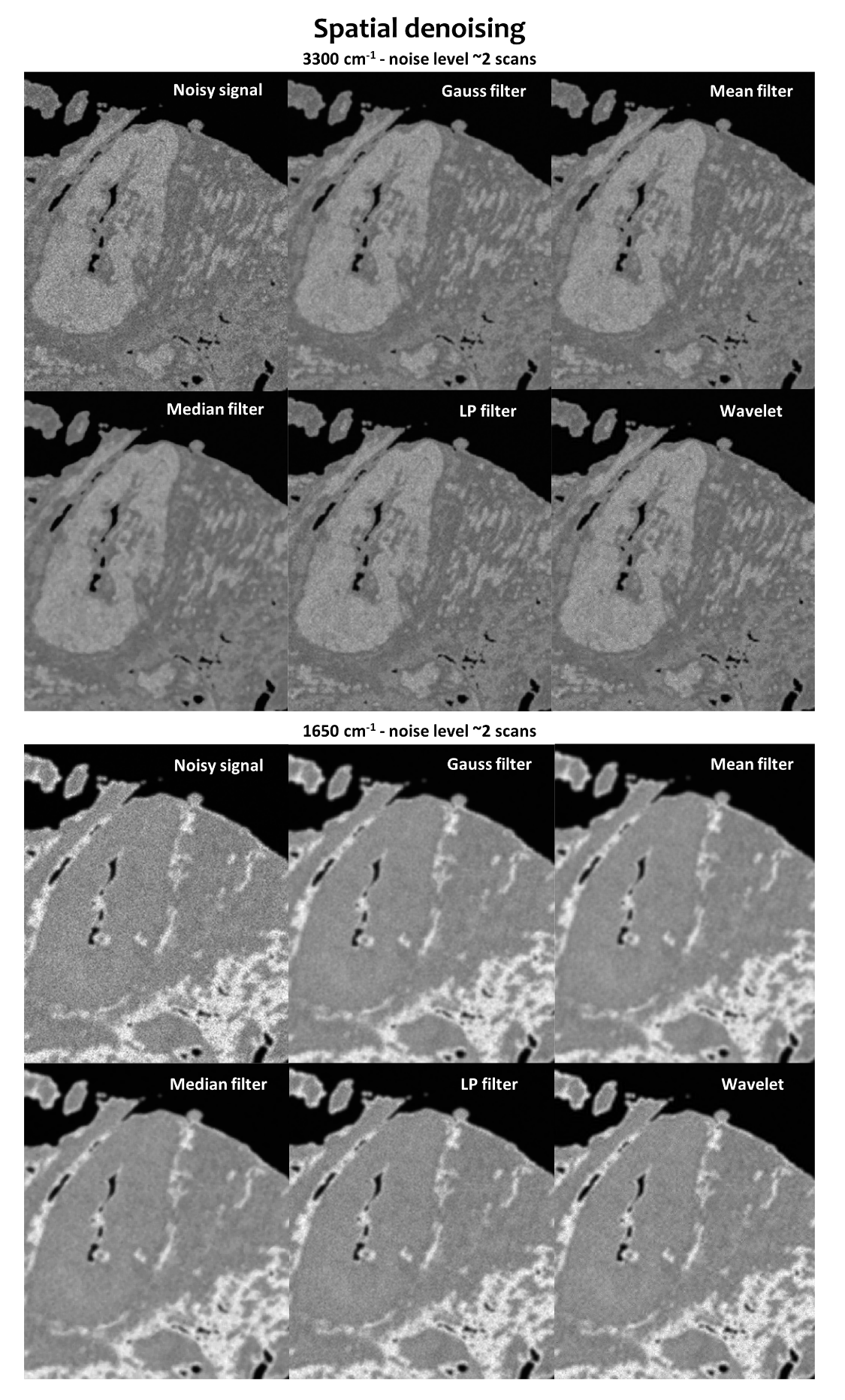


**S10.** Effects of spatial denoising of all techniques on the larger image shown in Figure 5, where only zooms are presented.

**
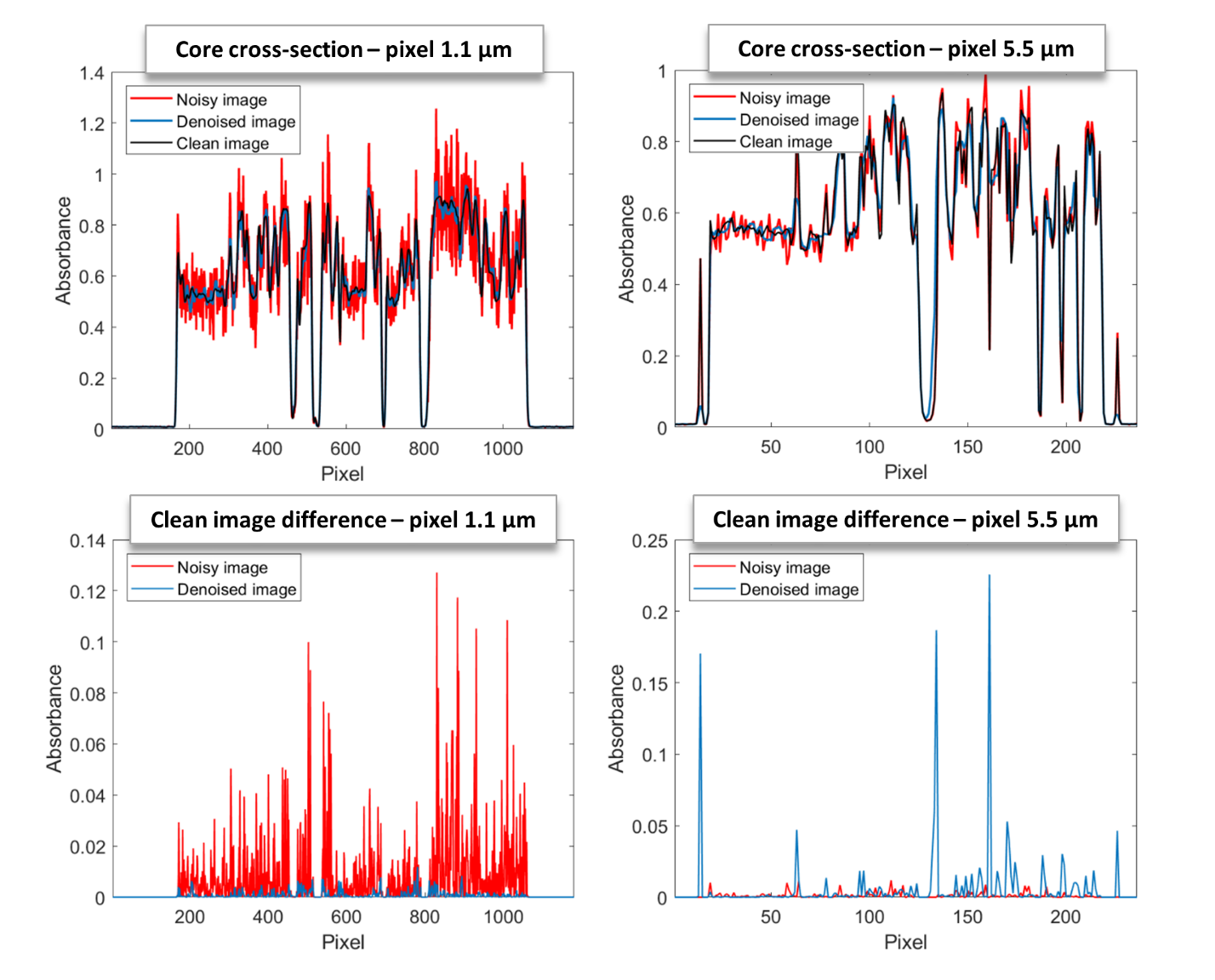
**

**S11.** (top) Core section analysis for 5.5 µm and 1.1 µm projected pixel size, presenting structure sampling. (bottom) Differences between noisy and clean, and denoised and clean signal (absolute values) for both cases showing structural distortion for 5.5 µm pixel size as a result of undersampling.

**
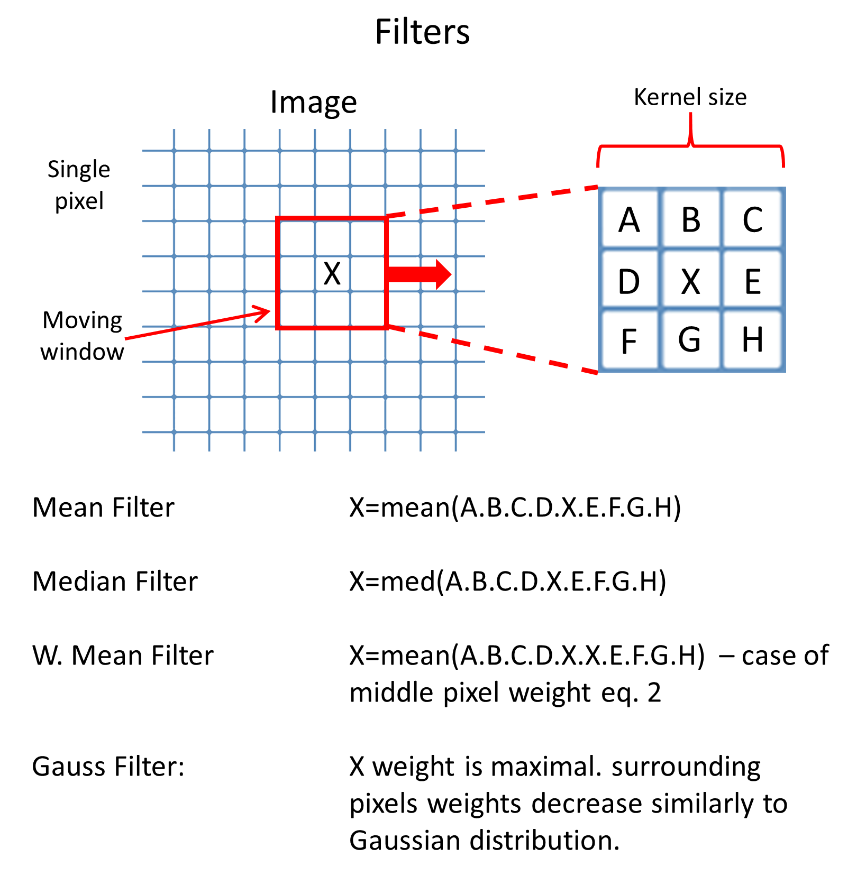
**

**S12.** Mechanism of different filters action (case of kernel size 3)**.**

**
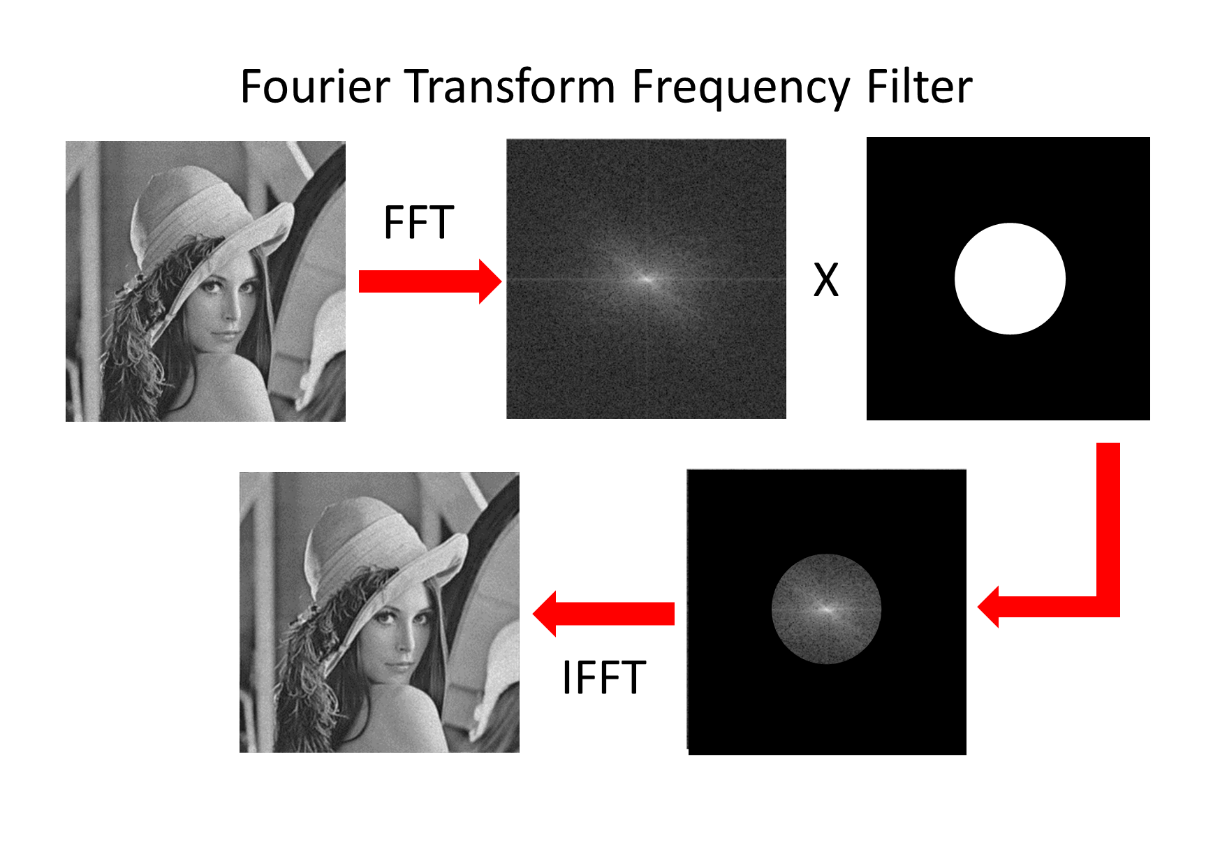
**

**S13.** Mechanism of Fourier Transform Frequency Filter**.**

**
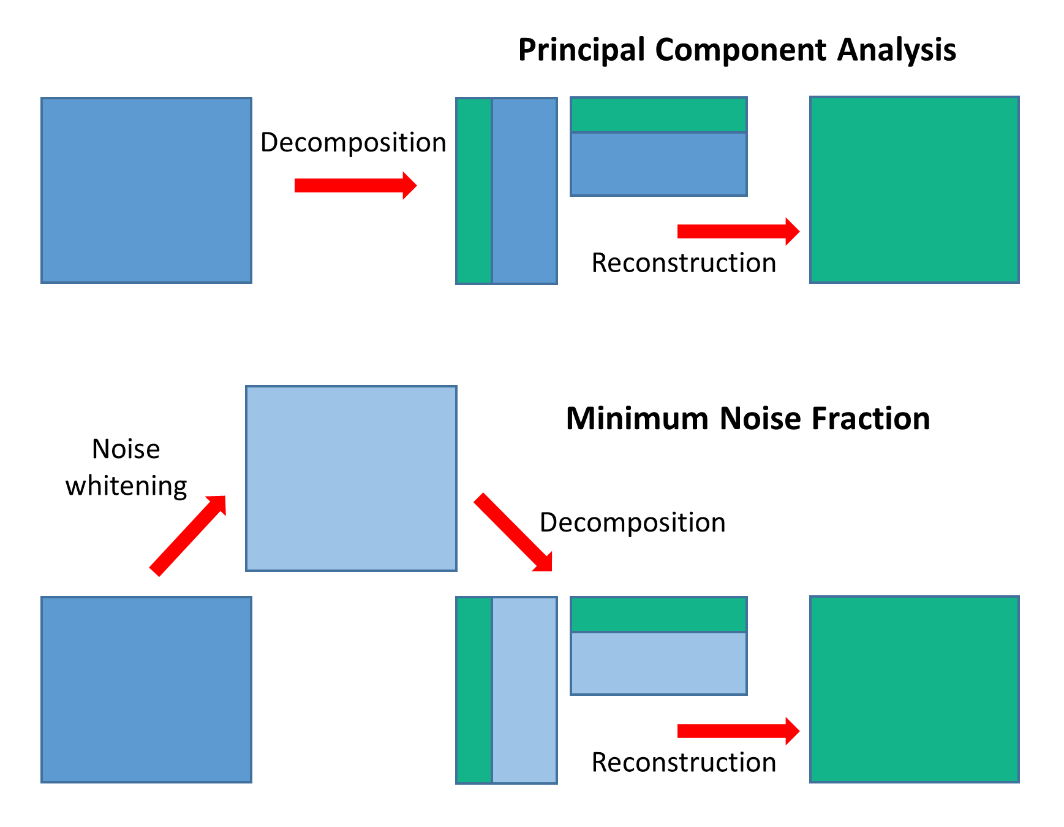
**

**S14.** Mechanism of PCA and MNF algorithms.

**
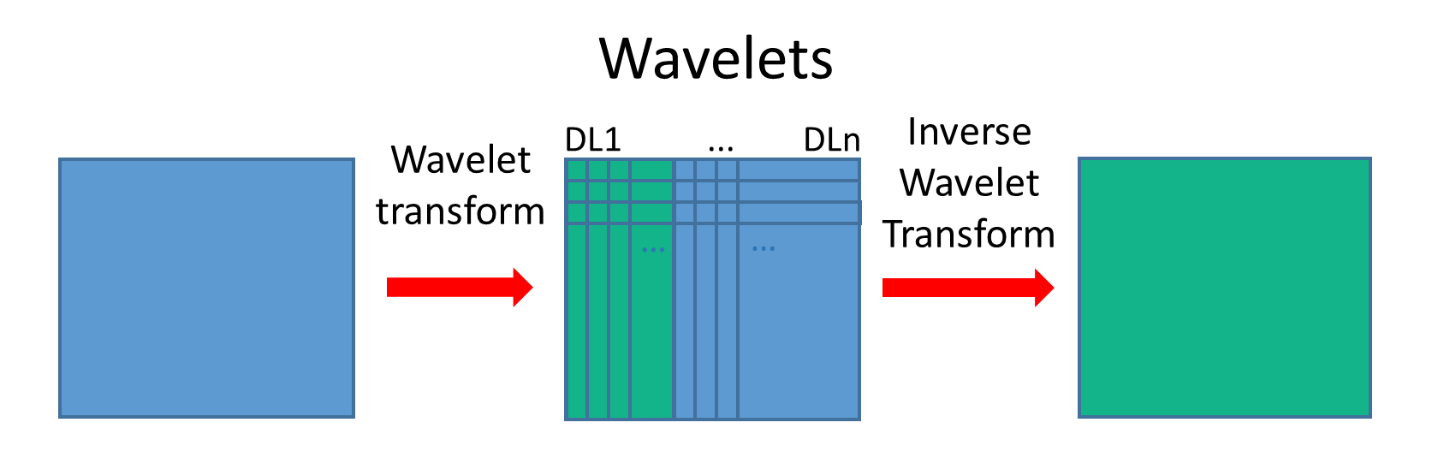
**

**S15.** Mechanism of Wavelet denoising algorithm.

*Wavelet denoising optimization*

As Wavelets do not have an optimization parameter that can be tuned, a set of 450 different families with different levels were tested and are given below. The final choice of the best method is somewhat arbitrary and was based on SNR gain and on Pearson correlation coefficient.

**Table 1**. Wavelets numbers with corresponding names and levels.

| **Wavelet no.** | **Wavelet name** |  | **Wavelet no.** | **Wavelet name** |  | **Wavelet no.** | **Wavelet name** |
| --- | --- | --- | --- | --- | --- | --- | --- |
| 1 | db1_1 |  | 51 | db6_6 |  | 101 | coif3_2 |
| 2 | db1_2 |  | 52 | db6_7 |  | 102 | coif3_3 |
| 3 | db1_3 |  | 53 | db6_8 |  | 103 | coif3_4 |
| 4 | db1_4 |  | 54 | db6_9 |  | 104 | coif3_5 |
| 5 | db1_5 |  | 55 | db7_1 |  | 105 | coif3_6 |
| 6 | db1_6 |  | 56 | db7_2 |  | 106 | coif3_7 |
| 7 | db1_7 |  | 57 | db7_3 |  | 107 | coif3_8 |
| 8 | db1_8 |  | 58 | db7_4 |  | 108 | coif3_9 |
| 9 | db1_9 |  | 59 | db7_5 |  | 109 | coif4_1 |
| 10 | db2_1 |  | 60 | db7_6 |  | 110 | coif4_2 |
| 11 | db2_2 |  | 61 | db7_7 |  | 111 | coif4_3 |
| 12 | db2_3 |  | 62 | db7_8 |  | 112 | coif4_4 |
| 13 | db2_4 |  | 63 | db7_9 |  | 113 | coif4_5 |
| 14 | db2_5 |  | 64 | db8_1 |  | 114 | coif4_6 |
| 15 | db2_6 |  | 65 | db8_2 |  | 115 | coif4_7 |
| 16 | db2_7 |  | 66 | db8_3 |  | 116 | coif4_8 |
| 17 | db2_8 |  | 67 | db8_4 |  | 117 | coif4_9 |
| 18 | db2_9 |  | 68 | db8_5 |  | 118 | coif5_1 |
| 19 | db3_1 |  | 69 | db8_6 |  | 119 | coif5_2 |
| 20 | db3_2 |  | 70 | db8_7 |  | 120 | coif5_3 |
| 21 | db3_3 |  | 71 | db8_8 |  | 121 | coif5_4 |
| 22 | db3_4 |  | 72 | db8_9 |  | 122 | coif5_5 |
| 23 | db3_5 |  | 73 | db9_1 |  | 123 | coif5_6 |
| 24 | db3_6 |  | 74 | db9_2 |  | 124 | coif5_7 |
| 25 | db3_7 |  | 75 | db9_3 |  | 125 | coif5_8 |
| 26 | db3_8 |  | 76 | db9_4 |  | 126 | coif5_9 |
| 27 | db3_9 |  | 77 | db9_5 |  | 127 | bior1.1_1 |
| 28 | db4_1 |  | 78 | db9_6 |  | 128 | bior1.1_2 |
| 29 | db4_2 |  | 79 | db9_7 |  | 129 | bior1.1_3 |
| 30 | db4_3 |  | 80 | db9_8 |  | 130 | bior1.1_4 |
| 31 | db4_4 |  | 81 | db9_9 |  | 131 | bior1.1_5 |
| 32 | db4_5 |  | 82 | coif1_1 |  | 132 | bior1.1_6 |
| 33 | db4_6 |  | 83 | coif1_2 |  | 133 | bior1.1_7 |
| 34 | db4_7 |  | 84 | coif1_3 |  | 134 | bior1.1_8 |
| 35 | db4_8 |  | 85 | coif1_4 |  | 135 | bior1.1_9 |
| 36 | db4_9 |  | 86 | coif1_5 |  | 136 | bior1.3_1 |
| 37 | db5_1 |  | 87 | coif1_6 |  | 137 | bior1.3_2 |
| 38 | db5_2 |  | 88 | coif1_7 |  | 138 | bior1.3_3 |
| 39 | db5_3 |  | 89 | coif1_8 |  | 139 | bior1.3_4 |
| 40 | db5_4 |  | 90 | coif1_9 |  | 140 | bior1.3_5 |
| 41 | db5_5 |  | 91 | coif2_1 |  | 141 | bior1.3_6 |
| 42 | db5_6 |  | 92 | coif2_2 |  | 142 | bior1.3_7 |
| 43 | db5_7 |  | 93 | coif2_3 |  | 143 | bior1.3_8 |
| 44 | db5_8 |  | 94 | coif2_4 |  | 144 | bior1.3_9 |
| 45 | db5_9 |  | 95 | coif2_5 |  | 145 | bior1.5_1 |
| 46 | db6_1 |  | 96 | coif2_6 |  | 146 | bior1.5_2 |
| 47 | db6_2 |  | 97 | coif2_7 |  | 147 | bior1.5_3 |
| 48 | db6_3 |  | 98 | coif2_8 |  | 148 | bior1.5_4 |
| 49 | db6_4 |  | 99 | coif2_9 |  | 149 | bior1.5_5 |
| 50 | db6_5 |  | 100 | coif3_1 |  | 150 | bior1.5_6 |

| **Wavelet no.** | **Wavelet name** |  | **Wavelet no.** | **Wavelet name** |  | **Wavelet no.** | **Wavelet name** |
| --- | --- | --- | --- | --- | --- | --- | --- |
| 151 | bior1.5_7 |  | 201 | bior3.7_3 |  | 251 | rbio1.1_8 |
| 152 | bior1.5_8 |  | 202 | bior3.7_4 |  | 252 | rbio1.1_9 |
| 153 | bior1.5_9 |  | 203 | bior3.7_5 |  | 253 | rbio1.3_1 |
| 154 | bior2.2_1 |  | 204 | bior3.7_6 |  | 254 | rbio1.3_2 |
| 155 | bior2.2_2 |  | 205 | bior3.7_7 |  | 255 | rbio1.3_3 |
| 156 | bior2.2_3 |  | 206 | bior3.7_8 |  | 256 | rbio1.3_4 |
| 157 | bior2.2_4 |  | 207 | bior3.7_9 |  | 257 | rbio1.3_5 |
| 158 | bior2.2_5 |  | 208 | bior3.9_1 |  | 258 | rbio1.3_6 |
| 159 | bior2.2_6 |  | 209 | bior3.9_2 |  | 259 | rbio1.3_7 |
| 160 | bior2.2_7 |  | 210 | bior3.9_3 |  | 260 | rbio1.3_8 |
| 161 | bior2.2_8 |  | 211 | bior3.9_4 |  | 261 | rbio1.3_9 |
| 162 | bior2.2_9 |  | 212 | bior3.9_5 |  | 262 | rbio1.5_1 |
| 163 | bior2.4_1 |  | 213 | bior3.9_6 |  | 263 | rbio1.5_2 |
| 164 | bior2.4_2 |  | 214 | bior3.9_7 |  | 264 | rbio1.5_3 |
| 165 | bior2.4_3 |  | 215 | bior3.9_8 |  | 265 | rbio1.5_4 |
| 166 | bior2.4_4 |  | 216 | bior3.9_9 |  | 266 | rbio1.5_5 |
| 167 | bior2.4_5 |  | 217 | bior4.4_1 |  | 267 | rbio1.5_6 |
| 168 | bior2.4_6 |  | 218 | bior4.4_2 |  | 268 | rbio1.5_7 |
| 169 | bior2.4_7 |  | 219 | bior4.4_3 |  | 269 | rbio1.5_8 |
| 170 | bior2.4_8 |  | 220 | bior4.4_4 |  | 270 | rbio1.5_9 |
| 171 | bior2.4_9 |  | 221 | bior4.4_5 |  | 271 | rbio2.2_1 |
| 172 | bior2.8_1 |  | 222 | bior4.4_6 |  | 272 | rbio2.2_2 |
| 173 | bior2.8_2 |  | 223 | bior4.4_7 |  | 273 | rbio2.2_3 |
| 174 | bior2.8_3 |  | 224 | bior4.4_8 |  | 274 | rbio2.2_4 |
| 175 | bior2.8_4 |  | 225 | bior4.4_9 |  | 275 | rbio2.2_5 |
| 176 | bior2.8_5 |  | 226 | bior5.5_1 |  | 276 | rbio2.2_6 |
| 177 | bior2.8_6 |  | 227 | bior5.5_2 |  | 277 | rbio2.2_7 |
| 178 | bior2.8_7 |  | 228 | bior5.5_3 |  | 278 | rbio2.2_8 |
| 179 | bior2.8_8 |  | 229 | bior5.5_4 |  | 279 | rbio2.2_9 |
| 180 | bior2.8_9 |  | 230 | bior5.5_5 |  | 280 | rbio2.4_1 |
| 181 | bior3.3_1 |  | 231 | bior5.5_6 |  | 281 | rbio2.4_2 |
| 182 | bior3.3_2 |  | 232 | bior5.5_7 |  | 282 | rbio2.4_3 |
| 183 | bior3.3_3 |  | 233 | bior5.5_8 |  | 283 | rbio2.4_4 |
| 184 | bior3.3_4 |  | 234 | bior5.5_9 |  | 284 | rbio2.4_5 |
| 185 | bior3.3_5 |  | 235 | bior6.8_1 |  | 285 | rbio2.4_6 |
| 186 | bior3.3_6 |  | 236 | bior6.8_2 |  | 286 | rbio2.4_7 |
| 187 | bior3.3_7 |  | 237 | bior6.8_3 |  | 287 | rbio2.4_8 |
| 188 | bior3.3_8 |  | 238 | bior6.8_4 |  | 288 | rbio2.4_9 |
| 189 | bior3.3_9 |  | 239 | bior6.8_5 |  | 289 | rbio2.8_1 |
| 190 | bior3.5_1 |  | 240 | bior6.8_6 |  | 290 | rbio2.8_2 |
| 191 | bior3.5_2 |  | 241 | bior6.8_7 |  | 291 | rbio2.8_3 |
| 192 | bior3.5_3 |  | 242 | bior6.8_8 |  | 292 | rbio2.8_4 |
| 193 | bior3.5_4 |  | 243 | bior6.8_9 |  | 293 | rbio2.8_5 |
| 194 | bior3.5_5 |  | 244 | rbio1.1_1 |  | 294 | rbio2.8_6 |
| 195 | bior3.5_6 |  | 245 | rbio1.1_2 |  | 295 | rbio2.8_7 |
| 196 | bior3.5_7 |  | 246 | rbio1.1_3 |  | 296 | rbio2.8_8 |
| 197 | bior3.5_8 |  | 247 | rbio1.1_4 |  | 297 | rbio2.8_9 |
| 198 | bior3.5_9 |  | 248 | rbio1.1_5 |  | 298 | rbio3.3_1 |
| 199 | bior3.7_1 |  | 249 | rbio1.1_6 |  | 299 | rbio3.3_2 |
| 200 | bior3.7_2 |  | 250 | rbio1.1_7 |  | 300 | rbio3.3_3 |

| **Wavelet no.** | **Wavelet name** |  | **Wavelet no.** | **Wavelet name** |  | **Wavelet no.** | **Wavelet name** |
| --- | --- | --- | --- | --- | --- | --- | --- |
| 301 | rbio3.3_4 |  | 351 | rbio5.5_9 |  | 401 | sym5_5 |
| 302 | rbio3.3_5 |  | 352 | rbio6.8_1 |  | 402 | sym5_6 |
| 303 | rbio3.3_6 |  | 353 | rbio6.8_2 |  | 403 | sym5_7 |
| 304 | rbio3.3_7 |  | 354 | rbio6.8_3 |  | 404 | sym5_8 |
| 305 | rbio3.3_8 |  | 355 | rbio6.8_4 |  | 405 | sym5_9 |
| 306 | rbio3.3_9 |  | 356 | rbio6.8_5 |  | 406 | sym6_1 |
| 307 | rbio3.5_1 |  | 357 | rbio6.8_6 |  | 407 | sym6_2 |
| 308 | rbio3.5_2 |  | 358 | rbio6.8_7 |  | 408 | sym6_3 |
| 309 | rbio3.5_3 |  | 359 | rbio6.8_8 |  | 409 | sym6_4 |
| 310 | rbio3.5_4 |  | 360 | rbio6.8_9 |  | 410 | sym6_5 |
| 311 | rbio3.5_5 |  | 361 | sym1_1 |  | 411 | sym6_6 |
| 312 | rbio3.5_6 |  | 362 | sym1_2 |  | 412 | sym6_7 |
| 313 | rbio3.5_7 |  | 363 | sym1_3 |  | 413 | sym6_8 |
| 314 | rbio3.5_8 |  | 364 | sym1_4 |  | 414 | sym6_9 |
| 315 | rbio3.5_9 |  | 365 | sym1_5 |  | 415 | sym7_1 |
| 316 | rbio3.7_1 |  | 366 | sym1_6 |  | 416 | sym7_2 |
| 317 | rbio3.7_2 |  | 367 | sym1_7 |  | 417 | sym7_3 |
| 318 | rbio3.7_3 |  | 368 | sym1_8 |  | 418 | sym7_4 |
| 319 | rbio3.7_4 |  | 369 | sym1_9 |  | 419 | sym7_5 |
| 320 | rbio3.7_5 |  | 370 | sym2_1 |  | 420 | sym7_6 |
| 321 | rbio3.7_6 |  | 371 | sym2_2 |  | 421 | sym7_7 |
| 322 | rbio3.7_7 |  | 372 | sym2_3 |  | 422 | sym7_8 |
| 323 | rbio3.7_8 |  | 373 | sym2_4 |  | 423 | sym7_9 |
| 324 | rbio3.7_9 |  | 374 | sym2_5 |  | 424 | sym8_1 |
| 325 | rbio3.9_1 |  | 375 | sym2_6 |  | 425 | sym8_2 |
| 326 | rbio3.9_2 |  | 376 | sym2_7 |  | 426 | sym8_3 |
| 327 | rbio3.9_3 |  | 377 | sym2_8 |  | 427 | sym8_4 |
| 328 | rbio3.9_4 |  | 378 | sym2_9 |  | 428 | sym8_5 |
| 329 | rbio3.9_5 |  | 379 | sym3_1 |  | 429 | sym8_6 |
| 330 | rbio3.9_6 |  | 380 | sym3_2 |  | 430 | sym8_7 |
| 331 | rbio3.9_7 |  | 381 | sym3_3 |  | 431 | sym8_8 |
| 332 | rbio3.9_8 |  | 382 | sym3_4 |  | 432 | sym8_9 |
| 333 | rbio3.9_9 |  | 383 | sym3_5 |  | 433 | dmey_1 |
| 334 | rbio4.4_1 |  | 384 | sym3_6 |  | 434 | dmey_2 |
| 335 | rbio4.4_2 |  | 385 | sym3_7 |  | 435 | dmey_3 |
| 336 | rbio4.4_3 |  | 386 | sym3_8 |  | 436 | dmey_4 |
| 337 | rbio4.4_4 |  | 387 | sym3_9 |  | 437 | dmey_5 |
| 338 | rbio4.4_5 |  | 388 | sym4_1 |  | 438 | dmey_6 |
| 339 | rbio4.4_6 |  | 389 | sym4_2 |  | 439 | dmey_7 |
| 340 | rbio4.4_7 |  | 390 | sym4_3 |  | 440 | dmey_8 |
| 341 | rbio4.4_8 |  | 391 | sym4_4 |  | 441 | dmey_9 |
| 342 | rbio4.4_9 |  | 392 | sym4_5 |  | 442 | haar_1 |
| 343 | rbio5.5_1 |  | 393 | sym4_6 |  | 443 | haar_2 |
| 344 | rbio5.5_2 |  | 394 | sym4_7 |  | 444 | haar_3 |
| 345 | rbio5.5_3 |  | 395 | sym4_8 |  | 445 | haar_4 |
| 346 | rbio5.5_4 |  | 396 | sym4_9 |  | 446 | haar_5 |
| 347 | rbio5.5_5 |  | 397 | sym5_1 |  | 447 | haar_6 |
| 348 | rbio5.5_6 |  | 398 | sym5_2 |  | 448 | haar_7 |
| 349 | rbio5.5_7 |  | 399 | sym5_3 |  | 449 | haar_8 |
| 350 | rbio5.5_8 |  | 400 | sym5_4 |  | 450 | haar_9 |

**Table 2.** Results of optimization of wavelet spectral denoising for projected pixel size of 1.1 µm and noise levels from 2-256 scans. Additional parameter, Pearson correlation coefficient R between denoised and clean signal, was introduced to avoid signal over-flattening. Wavelet with best performance is highlighted with blue.

| **Noise level ~2 scans** | | | |  | **Noise level ~4 scans** | | | |
| --- | --- | --- | --- | --- | --- | --- | --- | --- |
| **Wavelet** | **SNR Gain [a.u.]** | **SD [%]** | **R** |  | **Wavelet** | **SNR Gain [a.u.]** | **SD [%]** | **R** |
| db9_3 | 9,98 | 1,50 | 0,99038 |  | db9_3 | 9,47 | 1,09 | 0,99483 |
| db6_3 | 12,01 | 1,50 | 0,99025 |  | coif3_3 | 9,83 | 1,09 | 0,99479 |
| sym7_3 | 10,11 | 1,49 | 0,99024 |  | db6_3 | 10,12 | 1,10 | 0,99475 |
| coif3_3 | 9,72 | 1,49 | 0,99023 |  | sym7_3 | 8,91 | 1,11 | 0,9947 |
| coif2_3 | 9,90 | 1,51 | 0,99017 |  | coif2_3 | 9,61 | 1,11 | 0,99469 |
| coif4_3 | 10,36 | 1,52 | 0,99016 |  | dmey_3 | 9,20 | 1,13 | 0,99467 |
| dmey_3 | 9,12 | 1,52 | 0,99002 |  | bior6.8_3 | 8,83 | 1,14 | 0,99458 |
| rbio3.9_3 | 9,09 | 1,61 | 0,98992 |  | coif4_3 | 9,57 | 1,14 | 0,99457 |
| rbio6.8_3 | 9,32 | 1,56 | 0,98991 |  | rbio6.8_3 | 8,99 | 1,18 | 0,99449 |
| bior6.8_3 | 9,09 | 1,53 | 0,98985 |  | bior5.5_3 | 8,06 | 1,21 | 0,99444 |
| rbio2.8_3 | 9,43 | 1,60 | 0,98985 |  | bior4.4_3 | 7,69 | 1,17 | 0,99443 |
| db3_3 | 8,64 | 1,53 | 0,98982 |  | rbio2.8_3 | 8,26 | 1,22 | 0,99442 |
| sym3_3 | 8,64 | 1,53 | 0,98982 |  | db3_3 | 9,58 | 1,14 | 0,9944 |
| bior5.5_3 | 9,52 | 1,59 | 0,98976 |  | sym3_3 | 9,58 | 1,14 | 0,9944 |
| db7_3 | 8,85 | 1,57 | 0,98962 |  | rbio3.9_3 | 8,45 | 1,24 | 0,99419 |
| coif5_3 | 10,16 | 1,55 | 0,98962 |  | coif5_3 | 8,15 | 1,19 | 0,99417 |
| bior4.4_3 | 9,80 | 1,56 | 0,98955 |  | bior2.4_3 | 7,05 | 1,12 | 0,99408 |
| rbio5.5_4 | 33,00 | 1,63 | 0,98954 |  | sym8_3 | 9,38 | 1,22 | 0,99408 |
| sym8_3 | 9,70 | 1,59 | 0,98954 |  | bior2.8_3 | 8,26 | 1,12 | 0,99406 |
| sym7_4 | 31,87 | 1,79 | 0,98953 |  | sym6_3 | 9,02 | 1,22 | 0,99406 |
| sym6_3 | 9,56 | 1,59 | 0,98945 |  | rbio5.5_3 | 8,03 | 1,16 | 0,99396 |
| db4_3 | 8,47 | 1,57 | 0,98942 |  | db7_3 | 7,76 | 1,21 | 0,99395 |
| coif2_4 | 39,57 | 1,76 | 0,98934 |  | sym4_3 | 9,17 | 1,22 | 0,99395 |
| sym4_3 | 10,05 | 1,59 | 0,98928 |  | db4_3 | 7,03 | 1,22 | 0,99394 |
| db4_4 | 28,15 | 1,83 | 0,98922 |  | db8_3 | 8,80 | 1,25 | 0,99381 |
| sym5_3 | 8,41 | 1,60 | 0,98921 |  | rbio5.5_4 | 29,60 | 1,28 | 0,99379 |
| db8_3 | 9,34 | 1,61 | 0,9892 |  | bior2.2_3 | 6,23 | 1,18 | 0,99377 |
| db7_4 | 18,94 | 1,83 | 0,98917 |  | coif3_4 | 50,08 | 1,39 | 0,99374 |
| coif3_4 | 32,02 | 1,78 | 0,98917 |  | rbio4.4_3 | 7,63 | 1,26 | 0,99374 |
| coif4_4 | 36,12 | 1,80 | 0,98917 |  | rbio1.5_3 | 8,51 | 1,29 | 0,99372 |
| rbio3.7_3 | 8,67 | 1,68 | 0,98915 |  | coif2_4 | 30,86 | 1,40 | 0,99369 |
| rbio5.5_3 | 8,18 | 1,53 | 0,98903 |  | sym5_3 | 7,86 | 1,25 | 0,99364 |
| db3_4 | 23,81 | 1,80 | 0,98901 |  | rbio1.3_3 | 8,44 | 1,28 | 0,99362 |
| bior2.8_4 | 50,76 | 1,63 | 0,98901 |  | db5_3 | 8,76 | 1,28 | 0,99361 |
| sym3_4 | 23,81 | 1,80 | 0,98901 |  | bior2.8_4 | 50,19 | 1,28 | 0,99361 |
| rbio4.4_3 | 8,17 | 1,62 | 0,98897 |  | coif1_3 | 6,35 | 1,26 | 0,99356 |
| bior2.8_3 | 8,41 | 1,48 | 0,98892 |  | rbio3.7_3 | 8,10 | 1,32 | 0,99356 |
| db5_3 | 8,99 | 1,64 | 0,98889 |  | bior3.9_3 | 7,95 | 1,07 | 0,9934 |
| bior2.4_3 | 10,13 | 1,49 | 0,98888 |  | sym7_4 | 32,62 | 1,45 | 0,99338 |
| rbio1.3_3 | 8,28 | 1,65 | 0,98886 |  | bior2.4_4 | 36,74 | 1,32 | 0,99334 |
| sym4_4 | 33,37 | 1,81 | 0,98883 |  | coif4_4 | 45,32 | 1,45 | 0,99331 |
| rbio1.5_3 | 7,85 | 1,68 | 0,98882 |  | bior3.7_3 | 7,02 | 1,08 | 0,9933 |
| coif5_4 | 33,44 | 1,86 | 0,98874 |  | rbio2.4_3 | 7,21 | 1,36 | 0,99328 |
| bior2.4_4 | 48,61 | 1,65 | 0,98874 |  | db3_4 | 21,20 | 1,46 | 0,99319 |
| coif1_3 | 9,29 | 1,61 | 0,98871 |  | sym3_4 | 21,20 | 1,46 | 0,99319 |
| db8_4 | 21,70 | 1,92 | 0,98869 |  | bior3.9_4 | 20,13 | 1,18 | 0,99317 |
| rbio2.4_3 | 8,16 | 1,70 | 0,98863 |  | dmey_4 | 35,80 | 1,53 | 0,99314 |
| rbio5.5_5 | 50,07 | 1,89 | 0,9886 |  | bior6.8_4 | 43,23 | 1,48 | 0,99312 |
| db6_4 | 29,26 | 1,86 | 0,98854 |  | bior3.5_3 | 7,01 | 1,12 | 0,99305 |

| **Noise level ~8 scans** | | | |  | **Noise level ~16 scans** | | | |
| --- | --- | --- | --- | --- | --- | --- | --- | --- |
| **Wavelet** | **SNR Gain [a.u.]** | **SD [%]** | **R** |  | **Wavelet** | **SNR Gain [a.u.]** | **SD [%]** | **R** |
| coif3_3 | 10,41 | 0,88 | 0,9968 |  | coif3_3 | 8,73 | 0,71 | 0,99805 |
| db9_3 | 10,03 | 0,88 | 0,99673 |  | db6_3 | 8,09 | 0,73 | 0,998 |
| db6_3 | 10,63 | 0,89 | 0,9967 |  | db9_3 | 7,63 | 0,71 | 0,998 |
| dmey_3 | 8,48 | 0,93 | 0,99663 |  | dmey_3 | 7,51 | 0,77 | 0,9979 |
| coif2_3 | 9,56 | 0,91 | 0,99662 |  | coif2_3 | 7,60 | 0,75 | 0,99788 |
| sym7_3 | 9,24 | 0,90 | 0,99661 |  | sym7_3 | 8,51 | 0,75 | 0,99787 |
| coif4_3 | 8,52 | 0,93 | 0,99652 |  | coif4_3 | 8,61 | 0,77 | 0,99781 |
| bior6.8_3 | 9,16 | 0,95 | 0,99651 |  | bior6.8_3 | 7,83 | 0,78 | 0,99779 |
| rbio6.8_3 | 9,59 | 0,99 | 0,99645 |  | rbio6.8_3 | 7,98 | 0,82 | 0,99771 |
| bior5.5_3 | 11,38 | 1,01 | 0,99642 |  | bior5.5_3 | 7,93 | 0,85 | 0,99765 |
| db3_3 | 8,56 | 0,95 | 0,99636 |  | bior4.4_3 | 7,91 | 0,81 | 0,99764 |
| sym3_3 | 8,56 | 0,95 | 0,99636 |  | db3_3 | 7,28 | 0,80 | 0,99763 |
| bior4.4_3 | 10,55 | 0,98 | 0,99635 |  | sym3_3 | 7,28 | 0,80 | 0,99763 |
| rbio2.8_3 | 10,20 | 1,03 | 0,99634 |  | coif5_3 | 7,31 | 0,83 | 0,9976 |
| coif5_3 | 7,68 | 0,99 | 0,99621 |  | bior2.4_3 | 7,55 | 0,76 | 0,99756 |
| bior2.4_3 | 8,79 | 0,93 | 0,99615 |  | bior2.8_3 | 7,19 | 0,76 | 0,99756 |
| bior2.8_3 | 8,85 | 0,92 | 0,99615 |  | rbio2.8_3 | 7,97 | 0,87 | 0,99756 |
| rbio5.5_3 | 8,71 | 0,97 | 0,99608 |  | bior3.9_3 | 7,04 | 0,68 | 0,9975 |
| rbio3.9_3 | 9,05 | 1,04 | 0,99604 |  | rbio5.5_3 | 7,30 | 0,79 | 0,99749 |
| sym6_3 | 10,06 | 1,02 | 0,99603 |  | bior3.7_3 | 7,08 | 0,69 | 0,99746 |
| sym8_3 | 9,40 | 1,02 | 0,99603 |  | sym8_3 | 8,15 | 0,85 | 0,99743 |
| db7_3 | 7,40 | 1,01 | 0,99599 |  | bior2.2_3 | 6,88 | 0,80 | 0,99741 |
| sym4_3 | 10,23 | 1,03 | 0,99594 |  | db7_3 | 7,54 | 0,85 | 0,9974 |
| rbio5.5_4 | 23,70 | 1,06 | 0,99593 |  | sym6_3 | 8,42 | 0,85 | 0,9974 |
| bior2.2_3 | 9,03 | 0,98 | 0,99592 |  | rbio5.5_4 | 19,70 | 0,87 | 0,99739 |
| db4_3 | 7,10 | 1,01 | 0,9959 |  | bior3.5_3 | 6,74 | 0,71 | 0,99736 |
| rbio1.5_3 | 7,97 | 1,05 | 0,99587 |  | coif3_4 | 40,17 | 0,97 | 0,99734 |
| db8_3 | 8,77 | 1,05 | 0,99586 |  | bior2.8_4 | 27,73 | 0,87 | 0,99731 |
| bior3.9_3 | 6,50 | 0,85 | 0,99583 |  | sym4_3 | 8,58 | 0,86 | 0,9973 |
| coif3_4 | 41,42 | 1,17 | 0,99579 |  | db4_3 | 6,64 | 0,86 | 0,99728 |
| rbio4.4_3 | 8,68 | 1,07 | 0,99579 |  | db8_3 | 8,42 | 0,88 | 0,99728 |
| bior2.8_4 | 2249,58 | 1,05 | 0,99578 |  | rbio3.9_3 | 8,01 | 0,90 | 0,99727 |
| bior3.7_3 | 6,42 | 0,86 | 0,99578 |  | rbio1.5_3 | 7,40 | 0,88 | 0,99726 |
| db5_3 | 8,50 | 1,08 | 0,99569 |  | bior3.9_4 | 21,40 | 0,78 | 0,99724 |
| rbio1.3_3 | 8,47 | 1,06 | 0,99569 |  | coif5_2 | 2,93 | 0,67 | 0,9972 |
| bior3.5_3 | 6,41 | 0,88 | 0,99565 |  | db5_2 | 3,05 | 0,67 | 0,99719 |
| sym5_3 | 9,06 | 1,05 | 0,99565 |  | db7_2 | 2,98 | 0,67 | 0,99718 |
| coif2_4 | 36,24 | 1,18 | 0,99563 |  | coif2_2 | 2,91 | 0,66 | 0,99718 |
| bior3.9_4 | 21,01 | 0,95 | 0,99558 |  | coif3_2 | 2,90 | 0,67 | 0,99717 |
| bior2.4_4 | 49,46 | 1,09 | 0,99556 |  | rbio6.8_2 | 2,92 | 0,69 | 0,99717 |
| coif1_3 | 8,44 | 1,08 | 0,99555 |  | db3_2 | 2,83 | 0,67 | 0,99716 |
| bior3.7_4 | 20,34 | 0,97 | 0,9955 |  | db5_3 | 8,52 | 0,90 | 0,99716 |
| rbio3.7_3 | 8,65 | 1,14 | 0,99546 |  | coif2_4 | 22,75 | 0,99 | 0,99716 |
| bior3.9_5 | 22,68 | 1,01 | 0,99544 |  | bior3.7_4 | 20,86 | 0,79 | 0,99716 |
| sym7_4 | 30,54 | 1,22 | 0,99543 |  | bior6.8_2 | 2,94 | 0,68 | 0,99716 |
| dmey_4 | 30,68 | 1,30 | 0,99539 |  | rbio2.8_2 | 2,95 | 0,69 | 0,99716 |
| bior6.8_4 | 39,70 | 1,23 | 0,99538 |  | rbio4.4_3 | 7,33 | 0,90 | 0,99716 |
| bior3.7_5 | 27,04 | 1,03 | 0,99536 |  | sym3_2 | 2,83 | 0,67 | 0,99716 |
| coif4_4 | 42,93 | 1,23 | 0,99534 |  | sym7_2 | 2,96 | 0,68 | 0,99716 |

| **Noise level ~32 scans** | | | |  | **Noise level ~64 scans** | | | |
| --- | --- | --- | --- | --- | --- | --- | --- | --- |
| **Wavelet** | **SNR Gain [a.u.]** | **SD [%]** | **R** |  | **Wavelet** | **SNR Gain [a.u.]** | **SD [%]** | **R** |
| coif3_3 | 9,86 | 0,64 | 0,9985 |  | db9_3 | 8,11 | 0,59 | 0,99869 |
| db9_3 | 8,18 | 0,64 | 0,99848 |  | coif3_3 | 8,86 | 0,61 | 0,99869 |
| db6_3 | 8,73 | 0,66 | 0,99845 |  | db6_3 | 8,59 | 0,62 | 0,99864 |
| dmey_3 | 8,20 | 0,71 | 0,99836 |  | dmey_3 | 7,66 | 0,66 | 0,99858 |
| coif2_3 | 8,64 | 0,68 | 0,99834 |  | coif2_3 | 8,32 | 0,64 | 0,99854 |
| sym7_3 | 9,60 | 0,68 | 0,99833 |  | sym7_3 | 8,46 | 0,64 | 0,99853 |
| coif4_3 | 9,84 | 0,70 | 0,99829 |  | coif4_3 | 8,21 | 0,66 | 0,99849 |
| bior6.8_3 | 8,47 | 0,71 | 0,99826 |  | bior6.8_3 | 8,23 | 0,67 | 0,99849 |
| rbio6.8_3 | 8,52 | 0,74 | 0,99819 |  | rbio6.8_3 | 8,43 | 0,71 | 0,99841 |
| db3_3 | 7,85 | 0,71 | 0,99815 |  | bior3.9_3 | 7,15 | 0,57 | 0,99839 |
| sym3_3 | 7,85 | 0,71 | 0,99815 |  | coif5_2 | 3,20 | 0,52 | 0,99837 |
| bior3.9_3 | 7,28 | 0,60 | 0,99814 |  | db5_2 | 3,33 | 0,52 | 0,99836 |
| bior4.4_3 | 8,42 | 0,75 | 0,99813 |  | bior2.8_3 | 7,74 | 0,65 | 0,99836 |
| bior3.7_3 | 7,38 | 0,61 | 0,99812 |  | bior3.7_3 | 7,01 | 0,58 | 0,99836 |
| bior2.8_3 | 7,93 | 0,69 | 0,99811 |  | db3_3 | 7,69 | 0,68 | 0,99835 |
| bior2.4_3 | 7,97 | 0,69 | 0,9981 |  | db7_2 | 3,21 | 0,52 | 0,99835 |
| coif5_3 | 8,12 | 0,75 | 0,99809 |  | coif2_2 | 3,22 | 0,52 | 0,99835 |
| bior5.5_3 | 8,52 | 0,79 | 0,99809 |  | sym3_3 | 7,69 | 0,68 | 0,99835 |
| bior3.5_3 | 7,96 | 0,63 | 0,99806 |  | bior2.4_3 | 7,65 | 0,65 | 0,99834 |
| rbio5.5_3 | 7,62 | 0,71 | 0,99806 |  | bior4.4_3 | 8,33 | 0,71 | 0,99834 |
| db5_2 | 2,97 | 0,56 | 0,99803 |  | bior6.8_2 | 3,18 | 0,53 | 0,99834 |
| coif5_2 | 2,97 | 0,57 | 0,99803 |  | coif5_3 | 8,17 | 0,71 | 0,99833 |
| rbio2.8_3 | 8,54 | 0,80 | 0,99803 |  | rbio6.8_2 | 3,18 | 0,53 | 0,99833 |
| db7_2 | 3,04 | 0,57 | 0,99802 |  | dmey_2 | 3,19 | 0,54 | 0,99833 |
| coif2_2 | 2,89 | 0,56 | 0,99801 |  | coif3_2 | 3,13 | 0,53 | 0,99832 |
| coif3_2 | 3,05 | 0,58 | 0,998 |  | bior4.4_2 | 3,05 | 0,53 | 0,99832 |
| rbio6.8_2 | 2,95 | 0,58 | 0,998 |  | bior5.5_2 | 3,07 | 0,54 | 0,99832 |
| bior2.2_3 | 7,48 | 0,73 | 0,99799 |  | rbio2.8_2 | 3,14 | 0,54 | 0,99832 |
| bior6.8_2 | 2,99 | 0,58 | 0,99799 |  | rbio5.5_3 | 7,69 | 0,67 | 0,99832 |
| rbio2.8_2 | 2,92 | 0,59 | 0,99799 |  | sym7_2 | 3,17 | 0,53 | 0,99832 |
| sym7_2 | 2,99 | 0,58 | 0,99799 |  | db3_2 | 3,26 | 0,53 | 0,99831 |
| dmey_2 | 2,96 | 0,59 | 0,99799 |  | sym3_2 | 3,26 | 0,53 | 0,99831 |
| bior5.5_2 | 2,92 | 0,59 | 0,99798 |  | coif4_2 | 3,27 | 0,53 | 0,9983 |
| db3_2 | 2,85 | 0,57 | 0,99797 |  | bior3.5_3 | 6,30 | 0,60 | 0,9983 |
| coif4_2 | 2,90 | 0,58 | 0,99797 |  | bior5.5_3 | 8,78 | 0,75 | 0,9983 |
| bior4.4_2 | 2,90 | 0,58 | 0,99797 |  | db8_2 | 3,16 | 0,54 | 0,99828 |
| rbio5.5_4 | 25,25 | 0,78 | 0,99797 |  | db9_2 | 3,22 | 0,53 | 0,99827 |
| sym3_2 | 2,85 | 0,57 | 0,99797 |  | db6_2 | 3,20 | 0,53 | 0,99826 |
| sym8_3 | 9,31 | 0,77 | 0,99797 |  | bior2.4_2 | 2,96 | 0,52 | 0,99826 |
| db8_2 | 3,06 | 0,58 | 0,99795 |  | rbio3.9_2 | 3,27 | 0,56 | 0,99826 |
| coif3_4 | 36,98 | 0,87 | 0,99795 |  | db4_2 | 3,19 | 0,54 | 0,99825 |
| bior3.9_4 | 19,55 | 0,69 | 0,99795 |  | rbio2.8_3 | 8,70 | 0,76 | 0,99825 |
| rbio3.9_2 | 2,96 | 0,60 | 0,99795 |  | bior2.8_2 | 3,02 | 0,53 | 0,99824 |
| sym6_3 | 8,74 | 0,77 | 0,99795 |  | rbio5.5_2 | 2,99 | 0,54 | 0,99824 |
| db6_2 | 3,05 | 0,58 | 0,99794 |  | bior2.2_3 | 6,46 | 0,69 | 0,99823 |
| db9_2 | 3,01 | 0,59 | 0,99793 |  | rbio2.4_2 | 2,97 | 0,56 | 0,99823 |
| bior2.8_4 | 74,71 | 0,78 | 0,99793 |  | rbio3.7_2 | 3,28 | 0,57 | 0,99823 |
| db4_2 | 2,84 | 0,59 | 0,99792 |  | rbio4.4_2 | 2,98 | 0,55 | 0,99823 |
| bior2.4_2 | 2,82 | 0,56 | 0,99791 |  | sym5_2 | 3,14 | 0,54 | 0,99823 |

| **Noise level ~128 scans** | | | |  | **Noise level ~256 scans** | | | |
| --- | --- | --- | --- | --- | --- | --- | --- | --- |
| **Wavelet** | **SNR Gain [a.u.]** | **SD [%]** | **R** |  | **Wavelet** | **SNR Gain [a.u.]** | **SD [%]** | **R** |
| db9_3 | 8,96 | 0,49 | 0,99919 |  | db9_3 | 7,74 | 0,45 | 0,99932 |
| coif3_3 | 10,47 | 0,50 | 0,99915 |  | coif3_3 | 8,47 | 0,46 | 0,99927 |
| db6_3 | 9,40 | 0,51 | 0,99912 |  | db5_2 | 2,96 | 0,34 | 0,99926 |
| db5_2 | 3,10 | 0,38 | 0,9991 |  | coif5_2 | 2,91 | 0,35 | 0,99926 |
| coif5_2 | 2,92 | 0,39 | 0,9991 |  | db7_2 | 2,87 | 0,35 | 0,99925 |
| db7_2 | 2,96 | 0,39 | 0,99909 |  | coif2_2 | 2,88 | 0,35 | 0,99925 |
| coif2_2 | 3,01 | 0,39 | 0,99909 |  | coif3_2 | 2,91 | 0,35 | 0,99925 |
| bior6.8_2 | 2,90 | 0,39 | 0,99909 |  | coif4_2 | 2,85 | 0,36 | 0,99925 |
| dmey_3 | 7,68 | 0,54 | 0,99909 |  | bior4.4_2 | 2,88 | 0,35 | 0,99925 |
| coif3_2 | 3,19 | 0,39 | 0,99908 |  | bior6.8_2 | 2,92 | 0,35 | 0,99925 |
| coif4_2 | 2,99 | 0,40 | 0,99908 |  | db6_3 | 8,05 | 0,48 | 0,99924 |
| bior4.4_2 | 2,79 | 0,39 | 0,99908 |  | db9_2 | 2,99 | 0,35 | 0,99924 |
| bior5.5_2 | 2,81 | 0,40 | 0,99908 |  | bior5.5_2 | 2,89 | 0,36 | 0,99924 |
| rbio6.8_2 | 2,90 | 0,40 | 0,99908 |  | rbio6.8_2 | 2,93 | 0,36 | 0,99924 |
| dmey_2 | 2,96 | 0,40 | 0,99908 |  | sym7_2 | 2,87 | 0,36 | 0,99924 |
| sym7_2 | 3,12 | 0,40 | 0,99907 |  | dmey_2 | 2,92 | 0,36 | 0,99924 |
| db9_2 | 2,98 | 0,40 | 0,99906 |  | db8_2 | 2,93 | 0,36 | 0,99923 |
| rbio2.8_2 | 2,84 | 0,40 | 0,99906 |  | rbio2.8_2 | 2,94 | 0,36 | 0,99923 |
| db3_2 | 3,03 | 0,40 | 0,99905 |  | db6_2 | 2,94 | 0,36 | 0,99922 |
| db8_2 | 3,18 | 0,40 | 0,99905 |  | db3_2 | 2,85 | 0,36 | 0,99921 |
| sym3_2 | 3,03 | 0,40 | 0,99905 |  | db4_2 | 2,86 | 0,37 | 0,99921 |
| db4_2 | 2,92 | 0,40 | 0,99904 |  | sym3_2 | 2,85 | 0,36 | 0,99921 |
| db6_2 | 3,08 | 0,40 | 0,99904 |  | sym5_2 | 2,92 | 0,36 | 0,99921 |
| coif2_3 | 9,21 | 0,53 | 0,99904 |  | dmey_3 | 8,06 | 0,51 | 0,99921 |
| rbio3.9_2 | 3,19 | 0,41 | 0,99904 |  | bior2.4_2 | 2,82 | 0,35 | 0,9992 |
| bior2.4_2 | 2,73 | 0,39 | 0,99903 |  | rbio3.9_2 | 2,87 | 0,37 | 0,9992 |
| bior3.9_3 | 7,04 | 0,45 | 0,99903 |  | sym6_2 | 2,93 | 0,36 | 0,9992 |
| coif4_3 | 9,12 | 0,55 | 0,99902 |  | sym8_2 | 2,93 | 0,36 | 0,9992 |
| bior2.8_2 | 2,77 | 0,40 | 0,99902 |  | coif4_3 | 9,08 | 0,50 | 0,99919 |
| bior6.8_3 | 8,35 | 0,56 | 0,99902 |  | bior2.8_2 | 2,80 | 0,35 | 0,99919 |
| sym5_2 | 3,14 | 0,40 | 0,99902 |  | bior3.9_2 | 2,84 | 0,34 | 0,99919 |
| sym7_3 | 10,11 | 0,54 | 0,99902 |  | bior3.9_3 | 6,54 | 0,42 | 0,99919 |
| sym8_2 | 2,91 | 0,40 | 0,99902 |  | sym4_2 | 2,92 | 0,37 | 0,99919 |
| bior3.7_3 | 6,77 | 0,46 | 0,99901 |  | coif2_3 | 7,78 | 0,49 | 0,99918 |
| rbio5.5_2 | 2,72 | 0,41 | 0,99901 |  | bior2.2_2 | 2,74 | 0,36 | 0,99918 |
| sym6_2 | 2,86 | 0,40 | 0,99901 |  | bior3.7_2 | 2,84 | 0,34 | 0,99918 |
| bior2.2_2 | 2,56 | 0,40 | 0,999 |  | bior3.7_3 | 6,29 | 0,42 | 0,99918 |
| rbio3.7_2 | 3,20 | 0,43 | 0,999 |  | rbio5.5_2 | 2,75 | 0,36 | 0,99918 |
| rbio4.4_2 | 2,70 | 0,42 | 0,999 |  | sym7_3 | 8,33 | 0,49 | 0,99918 |
| sym4_2 | 2,78 | 0,41 | 0,999 |  | rbio3.7_2 | 2,85 | 0,39 | 0,99917 |
| bior3.9_2 | 3,03 | 0,38 | 0,99898 |  | rbio4.4_2 | 2,75 | 0,38 | 0,99917 |
| rbio2.4_2 | 2,70 | 0,43 | 0,99898 |  | bior3.5_2 | 2,83 | 0,35 | 0,99916 |
| bior3.5_3 | 6,19 | 0,48 | 0,99897 |  | bior6.8_3 | 8,41 | 0,51 | 0,99916 |
| bior3.7_2 | 3,01 | 0,38 | 0,99897 |  | bior3.5_3 | 5,79 | 0,44 | 0,99915 |
| bior2.8_3 | 7,53 | 0,53 | 0,99895 |  | rbio2.4_2 | 2,78 | 0,39 | 0,99915 |
| rbio6.8_3 | 8,07 | 0,58 | 0,99895 |  | rbio1.3_2 | 2,87 | 0,40 | 0,99913 |
| bior3.5_2 | 2,90 | 0,40 | 0,99894 |  | rbio1.5_2 | 2,73 | 0,40 | 0,99913 |
| rbio5.5_3 | 7,21 | 0,53 | 0,99894 |  | bior2.8_3 | 7,52 | 0,48 | 0,99911 |
| coif5_3 | 7,74 | 0,57 | 0,99893 |  | rbio5.5_3 | 7,48 | 0,49 | 0,99911 |

**Table 3.** Results of optimization of wavelet spectral denoising for projected pixel size of 5.5 µm and noise levels from 2-256 scans. Additional parameter, Pearson correlation coefficient R between denoised and clean signal, was introduced to avoid signal over-flattening. Wavelet with best performance is highlighted with blue

| **Noise level ~2 scans** | | | |  | **Noise level ~4 scans** | | | |
| --- | --- | --- | --- | --- | --- | --- | --- | --- |
| **Wavelet** | **SNR Gain [a.u.]** | **SD [%]** | **R** |  | **Wavelet** | **SNR Gain [a.u.]** | **SD [%]** | **R** |
| coif3_3 | 8,25 | 0,71 | 0,99806 |  | db9_3 | 8,04 | 0,56 | 0,99887 |
| db9_3 | 8,60 | 0,70 | 0,99802 |  | coif3_3 | 8,18 | 0,57 | 0,99885 |
| db6_3 | 9,35 | 0,72 | 0,99797 |  | db6_3 | 8,39 | 0,59 | 0,99881 |
| dmey_3 | 8,18 | 0,76 | 0,99795 |  | dmey_3 | 7,15 | 0,62 | 0,99878 |
| sym7_3 | 7,63 | 0,74 | 0,99787 |  | coif2_3 | 7,63 | 0,61 | 0,99871 |
| coif2_3 | 8,57 | 0,74 | 0,99786 |  | sym7_3 | 7,75 | 0,61 | 0,99871 |
| coif4_3 | 8,18 | 0,76 | 0,99784 |  | coif4_3 | 7,90 | 0,62 | 0,9987 |
| bior6.8_3 | 9,85 | 0,77 | 0,99784 |  | bior6.8_3 | 8,66 | 0,63 | 0,99869 |
| rbio6.8_3 | 10,16 | 0,81 | 0,99774 |  | db5_2 | 3,24 | 0,47 | 0,99865 |
| bior5.5_3 | 9,08 | 0,84 | 0,99768 |  | coif5_2 | 3,35 | 0,47 | 0,99864 |
| bior4.4_3 | 8,96 | 0,80 | 0,99767 |  | db7_2 | 3,18 | 0,48 | 0,99863 |
| coif5_3 | 8,33 | 0,82 | 0,99763 |  | coif2_2 | 3,22 | 0,47 | 0,99863 |
| bior2.8_3 | 9,50 | 0,75 | 0,99763 |  | bior6.8_2 | 3,15 | 0,48 | 0,99862 |
| db3_3 | 7,87 | 0,78 | 0,99762 |  | dmey_2 | 3,35 | 0,49 | 0,99862 |
| bior2.4_3 | 8,31 | 0,75 | 0,99762 |  | coif4_2 | 3,41 | 0,49 | 0,99861 |
| sym3_3 | 7,87 | 0,78 | 0,99762 |  | bior3.9_3 | 6,79 | 0,53 | 0,99861 |
| rbio2.8_3 | 9,08 | 0,86 | 0,9976 |  | bior4.4_2 | 2,99 | 0,48 | 0,99861 |
| rbio5.5_3 | 9,29 | 0,78 | 0,99755 |  | bior5.5_2 | 3,01 | 0,49 | 0,99861 |
| bior3.9_3 | 7,03 | 0,69 | 0,99753 |  | rbio2.8_2 | 3,02 | 0,50 | 0,99861 |
| bior3.7_3 | 6,97 | 0,70 | 0,99749 |  | rbio6.8_2 | 3,17 | 0,49 | 0,99861 |
| sym8_3 | 9,71 | 0,84 | 0,99748 |  | rbio6.8_3 | 8,58 | 0,67 | 0,99861 |
| bior2.2_3 | 7,32 | 0,79 | 0,99747 |  | coif3_2 | 3,17 | 0,48 | 0,9986 |
| sym6_3 | 9,74 | 0,84 | 0,99747 |  | db3_2 | 3,09 | 0,48 | 0,99859 |
| rbio5.5_4 | 24,00 | 0,86 | 0,99745 |  | bior3.7_3 | 7,02 | 0,54 | 0,99859 |
| db7_3 | 7,83 | 0,85 | 0,99744 |  | sym3_2 | 3,09 | 0,48 | 0,99859 |
| coif3_4 | 34,13 | 0,95 | 0,99743 |  | sym7_2 | 3,26 | 0,49 | 0,99859 |
| bior2.8_4 | 215,05 | 0,85 | 0,99741 |  | db8_2 | 3,31 | 0,49 | 0,99857 |
| bior3.5_3 | 6,83 | 0,72 | 0,9974 |  | bior2.8_3 | 8,18 | 0,61 | 0,99857 |
| sym4_3 | 9,48 | 0,85 | 0,99738 |  | db6_2 | 3,25 | 0,49 | 0,99856 |
| db4_3 | 7,34 | 0,84 | 0,99736 |  | db9_2 | 3,29 | 0,49 | 0,99856 |
| db8_3 | 9,93 | 0,88 | 0,99734 |  | bior2.4_3 | 8,67 | 0,61 | 0,99856 |
| bior3.9_4 | 22,15 | 0,78 | 0,99734 |  | bior4.4_3 | 8,84 | 0,67 | 0,99856 |
| rbio1.5_3 | 7,99 | 0,88 | 0,99729 |  | coif5_3 | 8,85 | 0,67 | 0,99855 |
| rbio3.9_3 | 8,49 | 0,90 | 0,99728 |  | bior2.4_2 | 2,91 | 0,47 | 0,99855 |
| db5_2 | 2,88 | 0,66 | 0,99727 |  | db4_2 | 3,61 | 0,49 | 0,99854 |
| coif2_4 | 29,48 | 0,96 | 0,99727 |  | bior3.5_3 | 6,07 | 0,55 | 0,99854 |
| coif5_2 | 2,83 | 0,66 | 0,99727 |  | rbio3.9_2 | 3,21 | 0,50 | 0,99854 |
| bior3.7_4 | 19,91 | 0,79 | 0,99727 |  | bior2.8_2 | 3,00 | 0,48 | 0,99853 |
| bior3.9_5 | 23,35 | 0,82 | 0,99726 |  | rbio4.4_2 | 2,93 | 0,50 | 0,99853 |
| db7_2 | 2,81 | 0,66 | 0,99725 |  | rbio5.5_2 | 3,00 | 0,49 | 0,99853 |
| coif3_2 | 3,01 | 0,66 | 0,99725 |  | rbio5.5_3 | 7,99 | 0,62 | 0,99853 |
| rbio4.4_3 | 8,61 | 0,88 | 0,99725 |  | db3_3 | 7,27 | 0,65 | 0,99852 |
| coif2_2 | 2,81 | 0,65 | 0,99724 |  | rbio2.4_2 | 2,85 | 0,51 | 0,99852 |
| dmey_2 | 2,85 | 0,68 | 0,99724 |  | sym3_3 | 7,27 | 0,65 | 0,99852 |
| bior2.4_4 | 82,88 | 0,89 | 0,99723 |  | sym4_2 | 3,97 | 0,49 | 0,99852 |
| bior6.8_2 | 2,84 | 0,67 | 0,99723 |  | sym6_2 | 3,66 | 0,49 | 0,99852 |
| rbio2.8_2 | 2,84 | 0,68 | 0,99723 |  | sym8_2 | 3,56 | 0,50 | 0,99852 |
| rbio6.8_2 | 2,81 | 0,68 | 0,99723 |  | bior2.2_2 | 2,86 | 0,49 | 0,99851 |
| sym7_2 | 3,15 | 0,66 | 0,99722 |  | rbio3.7_2 | 3,17 | 0,52 | 0,99851 |

| **Noise level ~8 scans** | | | |  | **Noise level ~16 scans** | | | |
| --- | --- | --- | --- | --- | --- | --- | --- | --- |
| **Wavelet** | **SNR Gain [a.u.]** | **SD [%]** | **R** |  | **Wavelet** | **SNR Gain [a.u.]** | **SD [%]** | **R** |
| db9_3 | 8,90 | 0,46 | 0,99932 |  | coif5_2 | 3,23 | 0,27 | 0,99956 |
| coif5_2 | 2,99 | 0,35 | 0,9993 |  | db5_2 | 3,28 | 0,28 | 0,99955 |
| db5_2 | 3,18 | 0,35 | 0,99929 |  | db7_2 | 3,26 | 0,28 | 0,99955 |
| db7_2 | 3,18 | 0,35 | 0,99929 |  | bior6.8_2 | 3,23 | 0,28 | 0,99955 |
| coif3_3 | 10,03 | 0,47 | 0,99929 |  | db9_2 | 3,25 | 0,28 | 0,99954 |
| bior5.5_2 | 3,26 | 0,36 | 0,99929 |  | coif2_2 | 3,15 | 0,28 | 0,99954 |
| bior6.8_2 | 3,17 | 0,35 | 0,99929 |  | coif3_2 | 3,06 | 0,28 | 0,99954 |
| coif3_2 | 3,01 | 0,36 | 0,99928 |  | coif4_2 | 3,22 | 0,28 | 0,99954 |
| coif4_2 | 2,74 | 0,35 | 0,99928 |  | bior4.4_2 | 3,19 | 0,28 | 0,99954 |
| bior4.4_2 | 3,26 | 0,35 | 0,99928 |  | bior5.5_2 | 3,20 | 0,29 | 0,99954 |
| rbio6.8_2 | 3,12 | 0,36 | 0,99928 |  | rbio6.8_2 | 3,17 | 0,28 | 0,99954 |
| dmey_2 | 3,02 | 0,36 | 0,99928 |  | sym7_2 | 3,08 | 0,28 | 0,99954 |
| coif2_2 | 3,12 | 0,35 | 0,99927 |  | dmey_2 | 3,26 | 0,29 | 0,99954 |
| rbio2.8_2 | 3,26 | 0,36 | 0,99927 |  | db6_2 | 3,28 | 0,28 | 0,99953 |
| db9_2 | 2,97 | 0,35 | 0,99926 |  | db8_2 | 3,14 | 0,29 | 0,99953 |
| sym7_2 | 3,18 | 0,35 | 0,99926 |  | rbio2.8_2 | 3,11 | 0,29 | 0,99953 |
| db6_2 | 2,80 | 0,36 | 0,99925 |  | db4_2 | 3,05 | 0,29 | 0,99952 |
| db6_3 | 9,46 | 0,48 | 0,99925 |  | db9_3 | 9,06 | 0,39 | 0,99952 |
| db8_2 | 3,03 | 0,36 | 0,99925 |  | sym5_2 | 3,00 | 0,29 | 0,99952 |
| dmey_3 | 7,23 | 0,51 | 0,99925 |  | bior3.9_2 | 2,97 | 0,27 | 0,99951 |
| db4_2 | 2,70 | 0,36 | 0,99924 |  | sym6_2 | 3,12 | 0,29 | 0,99951 |
| bior2.4_2 | 2,97 | 0,35 | 0,99924 |  | sym8_2 | 3,18 | 0,29 | 0,99951 |
| db3_2 | 3,06 | 0,36 | 0,99923 |  | db3_2 | 3,14 | 0,30 | 0,9995 |
| sym3_2 | 3,06 | 0,36 | 0,99923 |  | bior2.4_2 | 3,08 | 0,29 | 0,9995 |
| sym6_2 | 2,74 | 0,36 | 0,99923 |  | bior3.7_2 | 2,92 | 0,27 | 0,9995 |
| sym8_2 | 2,76 | 0,36 | 0,99923 |  | rbio3.9_2 | 3,15 | 0,30 | 0,9995 |
| bior2.8_2 | 2,90 | 0,36 | 0,99922 |  | rbio5.5_2 | 2,96 | 0,30 | 0,9995 |
| rbio3.9_2 | 3,24 | 0,37 | 0,99922 |  | sym3_2 | 3,14 | 0,30 | 0,9995 |
| rbio5.5_2 | 2,88 | 0,37 | 0,99922 |  | sym4_2 | 3,01 | 0,29 | 0,9995 |
| sym4_2 | 2,71 | 0,36 | 0,99922 |  | coif3_3 | 8,69 | 0,39 | 0,99949 |
| sym5_2 | 3,11 | 0,36 | 0,99922 |  | bior2.2_2 | 2,83 | 0,29 | 0,99949 |
| bior2.2_2 | 2,92 | 0,36 | 0,99921 |  | bior2.8_2 | 3,02 | 0,29 | 0,99949 |
| bior3.9_3 | 7,77 | 0,42 | 0,99921 |  | bior3.5_2 | 2,82 | 0,28 | 0,99949 |
| coif4_3 | 10,24 | 0,50 | 0,9992 |  | rbio3.7_2 | 3,14 | 0,32 | 0,99949 |
| bior3.7_2 | 3,10 | 0,34 | 0,9992 |  | rbio4.4_2 | 2,95 | 0,31 | 0,99948 |
| bior3.7_3 | 7,62 | 0,42 | 0,9992 |  | db6_3 | 9,09 | 0,42 | 0,99947 |
| bior3.9_2 | 3,16 | 0,34 | 0,9992 |  | bior3.7_3 | 6,98 | 0,35 | 0,99947 |
| rbio3.7_2 | 3,23 | 0,38 | 0,9992 |  | bior3.9_3 | 6,92 | 0,35 | 0,99947 |
| rbio4.4_2 | 2,99 | 0,38 | 0,9992 |  | dmey_3 | 8,72 | 0,43 | 0,99947 |
| sym7_3 | 10,43 | 0,49 | 0,9992 |  | rbio1.5_2 | 2,85 | 0,32 | 0,99946 |
| coif2_3 | 9,46 | 0,50 | 0,99919 |  | rbio2.4_2 | 2,94 | 0,33 | 0,99946 |
| bior6.8_3 | 9,08 | 0,52 | 0,99919 |  | bior3.5_3 | 7,25 | 0,37 | 0,99945 |
| rbio2.4_2 | 3,13 | 0,39 | 0,99918 |  | rbio1.3_2 | 3,02 | 0,33 | 0,99945 |
| bior3.5_2 | 3,01 | 0,35 | 0,99917 |  | coif4_3 | 8,31 | 0,42 | 0,99944 |
| bior3.5_3 | 7,67 | 0,44 | 0,99916 |  | bior3.3_2 | 2,64 | 0,31 | 0,99944 |
| rbio1.3_2 | 3,36 | 0,40 | 0,99914 |  | sym7_3 | 8,13 | 0,42 | 0,99944 |
| bior2.8_3 | 7,92 | 0,48 | 0,99913 |  | bior6.8_3 | 9,90 | 0,43 | 0,99943 |
| rbio1.5_2 | 3,23 | 0,41 | 0,99913 |  | coif2_3 | 9,92 | 0,43 | 0,99942 |
| rbio5.5_3 | 7,76 | 0,49 | 0,99913 |  | bior2.8_3 | 8,90 | 0,40 | 0,99942 |

| **Noise level ~32 scans** | | | |  | **Noise level ~64 scans** | | | |
| --- | --- | --- | --- | --- | --- | --- | --- | --- |
| **Wavelet** | **SNR Gain [a.u.]** | **SD [%]** | **R** |  | **Wavelet** | **SNR Gain [a.u.]** | **SD [%]** | **R** |
| db5_2 | 3,16 | 0,22 | 0,99974 |  | coif5_2 | 2,94 | 0,18 | 0,99983 |
| coif3_2 | 3,36 | 0,22 | 0,99974 |  | db5_2 | 2,93 | 0,19 | 0,99982 |
| coif4_2 | 3,23 | 0,22 | 0,99974 |  | db7_2 | 2,95 | 0,19 | 0,99982 |
| coif5_2 | 3,13 | 0,21 | 0,99974 |  | db8_2 | 3,32 | 0,18 | 0,99982 |
| bior5.5_2 | 2,89 | 0,22 | 0,99974 |  | db9_2 | 2,92 | 0,19 | 0,99982 |
| bior6.8_2 | 3,06 | 0,21 | 0,99974 |  | coif3_2 | 3,25 | 0,18 | 0,99982 |
| sym7_2 | 3,31 | 0,22 | 0,99974 |  | coif4_2 | 2,98 | 0,18 | 0,99982 |
| db6_2 | 3,40 | 0,22 | 0,99973 |  | bior3.9_2 | 3,07 | 0,17 | 0,99982 |
| db7_2 | 3,11 | 0,22 | 0,99973 |  | bior4.4_2 | 2,87 | 0,19 | 0,99982 |
| db8_2 | 3,42 | 0,22 | 0,99973 |  | bior5.5_2 | 2,88 | 0,19 | 0,99982 |
| db9_2 | 3,15 | 0,22 | 0,99973 |  | bior6.8_2 | 2,92 | 0,18 | 0,99982 |
| bior4.4_2 | 2,85 | 0,22 | 0,99973 |  | rbio6.8_2 | 2,91 | 0,19 | 0,99982 |
| rbio2.8_2 | 2,96 | 0,23 | 0,99973 |  | sym7_2 | 3,18 | 0,18 | 0,99982 |
| rbio6.8_2 | 3,09 | 0,22 | 0,99973 |  | dmey_2 | 2,99 | 0,19 | 0,99982 |
| dmey_2 | 3,21 | 0,23 | 0,99973 |  | db6_2 | 3,28 | 0,18 | 0,99981 |
| db4_2 | 3,12 | 0,23 | 0,99972 |  | coif2_2 | 2,91 | 0,19 | 0,99981 |
| coif2_2 | 3,08 | 0,22 | 0,99972 |  | bior3.5_2 | 2,88 | 0,17 | 0,99981 |
| bior3.7_2 | 3,09 | 0,20 | 0,99972 |  | bior3.7_2 | 3,01 | 0,17 | 0,99981 |
| bior3.9_2 | 3,18 | 0,20 | 0,99972 |  | rbio2.8_2 | 2,87 | 0,19 | 0,99981 |
| sym5_2 | 3,36 | 0,22 | 0,99972 |  | sym5_2 | 3,11 | 0,19 | 0,99981 |
| sym8_2 | 3,18 | 0,23 | 0,99972 |  | sym8_2 | 2,92 | 0,19 | 0,99981 |
| bior3.5_2 | 2,98 | 0,21 | 0,99971 |  | db4_2 | 2,96 | 0,20 | 0,9998 |
| sym6_2 | 3,08 | 0,23 | 0,99971 |  | sym6_2 | 2,89 | 0,19 | 0,9998 |
| bior2.4_2 | 2,76 | 0,23 | 0,9997 |  | bior3.3_2 | 2,56 | 0,20 | 0,99979 |
| rbio3.9_2 | 3,32 | 0,24 | 0,9997 |  | rbio3.9_2 | 3,08 | 0,20 | 0,99979 |
| rbio5.5_2 | 2,90 | 0,24 | 0,9997 |  | rbio5.5_2 | 2,74 | 0,21 | 0,99979 |
| sym4_2 | 2,95 | 0,24 | 0,9997 |  | sym4_2 | 2,85 | 0,20 | 0,99979 |
| db3_2 | 3,04 | 0,24 | 0,99969 |  | db3_2 | 2,90 | 0,21 | 0,99978 |
| bior2.2_2 | 2,61 | 0,24 | 0,99969 |  | bior2.2_2 | 2,66 | 0,21 | 0,99978 |
| bior2.8_2 | 2,92 | 0,23 | 0,99969 |  | bior2.4_2 | 2,82 | 0,20 | 0,99978 |
| rbio1.5_2 | 2,88 | 0,25 | 0,99969 |  | bior2.8_2 | 2,79 | 0,20 | 0,99978 |
| sym3_2 | 3,04 | 0,24 | 0,99969 |  | rbio1.5_2 | 2,68 | 0,21 | 0,99978 |
| db9_3 | 9,67 | 0,33 | 0,99968 |  | sym3_2 | 2,90 | 0,21 | 0,99978 |
| bior3.3_2 | 2,74 | 0,23 | 0,99968 |  | bior3.9_3 | 7,76 | 0,24 | 0,99977 |
| rbio3.7_2 | 3,31 | 0,25 | 0,99968 |  | rbio3.7_2 | 3,06 | 0,22 | 0,99977 |
| rbio4.4_2 | 2,86 | 0,25 | 0,99968 |  | rbio4.4_2 | 2,74 | 0,22 | 0,99977 |
| coif3_3 | 10,76 | 0,32 | 0,99967 |  | db9_3 | 8,12 | 0,29 | 0,99976 |
| bior3.7_3 | 8,25 | 0,28 | 0,99967 |  | coif3_3 | 9,52 | 0,28 | 0,99976 |
| bior3.9_3 | 7,40 | 0,28 | 0,99967 |  | bior3.7_3 | 7,66 | 0,24 | 0,99976 |
| rbio1.3_2 | 3,03 | 0,26 | 0,99966 |  | rbio1.3_2 | 2,81 | 0,23 | 0,99976 |
| rbio2.4_2 | 2,80 | 0,27 | 0,99966 |  | bior3.5_3 | 7,07 | 0,25 | 0,99975 |
| dmey_3 | 7,38 | 0,36 | 0,99966 |  | rbio2.4_2 | 2,74 | 0,24 | 0,99975 |
| db6_3 | 10,46 | 0,34 | 0,99965 |  | db6_3 | 8,75 | 0,30 | 0,99974 |
| bior3.5_3 | 7,09 | 0,29 | 0,99965 |  | coif4_3 | 9,24 | 0,29 | 0,99974 |
| coif4_3 | 9,36 | 0,34 | 0,99964 |  | bior3.9_4 | 16,86 | 0,27 | 0,99974 |
| sym7_3 | 9,47 | 0,34 | 0,99964 |  | sym7_3 | 9,82 | 0,29 | 0,99974 |
| coif5_3 | 9,13 | 0,35 | 0,99963 |  | dmey_3 | 6,84 | 0,32 | 0,99974 |
| bior3.9_4 | 16,84 | 0,31 | 0,99963 |  | coif5_3 | 8,57 | 0,31 | 0,99973 |
| bior6.8_3 | 10,24 | 0,35 | 0,99963 |  | bior3.7_4 | 15,84 | 0,28 | 0,99973 |

| **Noise level ~128 scans** | | | |  | **Noise level ~256 scans** | | | |
| --- | --- | --- | --- | --- | --- | --- | --- | --- |
| **Wavelet** | **SNR Gain [a.u.]** | **SD [%]** | **R** |  | **Wavelet** | **SNR Gain [a.u.]** | **SD [%]** | **R** |
| coif3_2 | 2,92 | 0,16 | 0,99987 |  | coif5_2 | 3,29 | 0,15 | 0,99989 |
| coif5_2 | 3,11 | 0,16 | 0,99987 |  | db8_2 | 3,13 | 0,15 | 0,99988 |
| bior6.8_2 | 3,07 | 0,16 | 0,99987 |  | db9_2 | 3,20 | 0,15 | 0,99988 |
| db5_2 | 3,16 | 0,17 | 0,99986 |  | coif3_2 | 3,06 | 0,15 | 0,99988 |
| db6_2 | 2,93 | 0,16 | 0,99986 |  | coif4_2 | 3,18 | 0,15 | 0,99988 |
| db7_2 | 3,08 | 0,17 | 0,99986 |  | bior3.7_2 | 3,03 | 0,14 | 0,99988 |
| db8_2 | 2,93 | 0,16 | 0,99986 |  | bior3.9_2 | 3,04 | 0,13 | 0,99988 |
| db9_2 | 3,24 | 0,16 | 0,99986 |  | bior5.5_2 | 3,35 | 0,16 | 0,99988 |
| coif4_2 | 2,98 | 0,16 | 0,99986 |  | bior6.8_2 | 3,29 | 0,15 | 0,99988 |
| bior3.5_2 | 3,07 | 0,15 | 0,99986 |  | rbio6.8_2 | 3,28 | 0,16 | 0,99988 |
| bior3.7_2 | 3,10 | 0,15 | 0,99986 |  | sym7_2 | 3,08 | 0,15 | 0,99988 |
| bior3.9_2 | 3,07 | 0,15 | 0,99986 |  | db5_2 | 3,16 | 0,16 | 0,99987 |
| bior4.4_2 | 2,97 | 0,16 | 0,99986 |  | db6_2 | 3,10 | 0,15 | 0,99987 |
| bior5.5_2 | 2,98 | 0,17 | 0,99986 |  | db7_2 | 3,29 | 0,16 | 0,99987 |
| rbio2.8_2 | 2,99 | 0,17 | 0,99986 |  | coif2_2 | 3,02 | 0,16 | 0,99987 |
| rbio6.8_2 | 3,08 | 0,17 | 0,99986 |  | bior3.5_2 | 2,97 | 0,14 | 0,99987 |
| sym5_2 | 3,03 | 0,17 | 0,99986 |  | bior4.4_2 | 3,35 | 0,16 | 0,99987 |
| sym7_2 | 3,00 | 0,16 | 0,99986 |  | rbio2.8_2 | 3,28 | 0,16 | 0,99987 |
| dmey_2 | 3,14 | 0,17 | 0,99986 |  | sym5_2 | 3,09 | 0,16 | 0,99987 |
| coif2_2 | 3,06 | 0,17 | 0,99985 |  | sym6_2 | 3,22 | 0,16 | 0,99987 |
| sym6_2 | 2,98 | 0,17 | 0,99985 |  | sym8_2 | 3,22 | 0,16 | 0,99987 |
| sym8_2 | 3,00 | 0,17 | 0,99985 |  | dmey_2 | 3,29 | 0,16 | 0,99987 |
| db4_2 | 2,90 | 0,18 | 0,99984 |  | db4_2 | 3,20 | 0,17 | 0,99986 |
| bior3.3_2 | 2,91 | 0,17 | 0,99984 |  | bior3.3_2 | 2,86 | 0,16 | 0,99986 |
| rbio1.5_2 | 2,99 | 0,19 | 0,99984 |  | bior2.4_2 | 3,28 | 0,17 | 0,99985 |
| rbio3.9_2 | 3,07 | 0,18 | 0,99984 |  | bior2.8_2 | 3,15 | 0,17 | 0,99985 |
| db3_2 | 3,00 | 0,19 | 0,99983 |  | rbio1.5_2 | 3,05 | 0,18 | 0,99985 |
| bior2.2_2 | 2,80 | 0,19 | 0,99983 |  | rbio3.9_2 | 3,18 | 0,17 | 0,99985 |
| bior2.4_2 | 2,92 | 0,18 | 0,99983 |  | rbio5.5_2 | 3,10 | 0,17 | 0,99985 |
| bior2.8_2 | 2,95 | 0,18 | 0,99983 |  | sym4_2 | 3,17 | 0,17 | 0,99985 |
| rbio5.5_2 | 2,92 | 0,18 | 0,99983 |  | db3_2 | 2,95 | 0,18 | 0,99984 |
| sym3_2 | 3,00 | 0,19 | 0,99983 |  | bior2.2_2 | 3,28 | 0,18 | 0,99984 |
| sym4_2 | 2,93 | 0,18 | 0,99983 |  | bior3.7_3 | 7,36 | 0,20 | 0,99984 |
| bior3.7_3 | 6,90 | 0,22 | 0,99982 |  | bior3.9_3 | 7,40 | 0,20 | 0,99984 |
| bior3.9_3 | 7,36 | 0,21 | 0,99982 |  | sym3_2 | 2,95 | 0,18 | 0,99984 |
| rbio1.3_2 | 3,13 | 0,20 | 0,99982 |  | coif3_3 | 8,60 | 0,24 | 0,99983 |
| rbio3.7_2 | 3,09 | 0,20 | 0,99982 |  | bior3.5_3 | 6,68 | 0,21 | 0,99983 |
| rbio4.4_2 | 2,90 | 0,20 | 0,99982 |  | rbio1.3_2 | 3,22 | 0,19 | 0,99983 |
| db9_3 | 8,97 | 0,26 | 0,99981 |  | rbio3.7_2 | 3,20 | 0,19 | 0,99983 |
| coif3_3 | 9,08 | 0,25 | 0,99981 |  | rbio4.4_2 | 3,09 | 0,19 | 0,99983 |
| bior3.5_3 | 6,45 | 0,23 | 0,99981 |  | db9_3 | 9,36 | 0,25 | 0,99982 |
| coif4_3 | 9,24 | 0,27 | 0,9998 |  | coif4_3 | 7,93 | 0,25 | 0,99982 |
| bior3.9_4 | 18,01 | 0,24 | 0,9998 |  | coif5_3 | 8,57 | 0,25 | 0,99982 |
| rbio2.4_2 | 2,86 | 0,22 | 0,9998 |  | bior3.9_4 | 19,44 | 0,22 | 0,99982 |
| sym7_3 | 10,23 | 0,26 | 0,9998 |  | rbio2.4_2 | 3,09 | 0,21 | 0,99982 |
| dmey_3 | 6,41 | 0,29 | 0,9998 |  | sym7_3 | 8,00 | 0,25 | 0,99982 |
| db3_1 | 1,47 | 0,13 | 0,99979 |  | dmey_3 | 6,66 | 0,27 | 0,99982 |
| db4_1 | 1,61 | 0,13 | 0,99979 |  | db3_1 | 1,57 | 0,12 | 0,99981 |
| db5_1 | 1,56 | 0,13 | 0,99979 |  | db4_1 | 1,68 | 0,12 | 0,99981 |
